# Network tailoring of organosilica membranes *via* aluminum doping to improve the humid-gas separation performance†

**DOI:** 10.1039/d1ra07866f

**Published:** 2022-02-16

**Authors:** Norihiro Moriyama, Misato Ike, Hiroki Nagasawa, Masakoto Kanezashi, Toshinori Tsuru

**Affiliations:** Department of Chemical Engineering, Hiroshima University 1-4-1 Kagami-yama Higashi-Hiroshima 739-8527 Japan tsuru@hiroshima-u.ac.jp

## Abstract

Organosilica membranes have recently attracted much attention due to excellent hydrothermal stability which enables their use in the presence of water. In particular, during humid-gas separations at moderate-to-high temperatures, these membranes have shown excellent water permeance and moderate water selectivity, which has been a breakthrough in separation performance. In the present work, we found that aluminum doping into the bis(triethoxysilyl)ethane (BTESE)-derived organosilica structure further improves water selectivity (H_2_O/N_2_, H_2_O/H_2_) while maintaining a level of water permeance that reaches as high as several 10^−6^ mol (m^−2^ s^−1^ Pa^−1^). Single-gas permeation and nitrogen adsorption experiments have revealed that aluminum doping promotes densification of the pore structure and improves molecular sieving. In addition, water adsorption and desorption experiments have revealed that aluminum doping enhances water adsorption onto the pore walls, which blocks permeation by other gasses and significantly improves water permeation selectivity during the separation of humid gases. Our results provide a strategy for the fabrication of a membrane that provides both a high level of water permeance and enhanced water selectivity.

## Abstract 2

Al doping densified and hydrophilized the pore structure of organosilica membranes, which resulted in improved permselectivity in humid-gas separation at moderate-to-high temperature.

## Graphical abstract

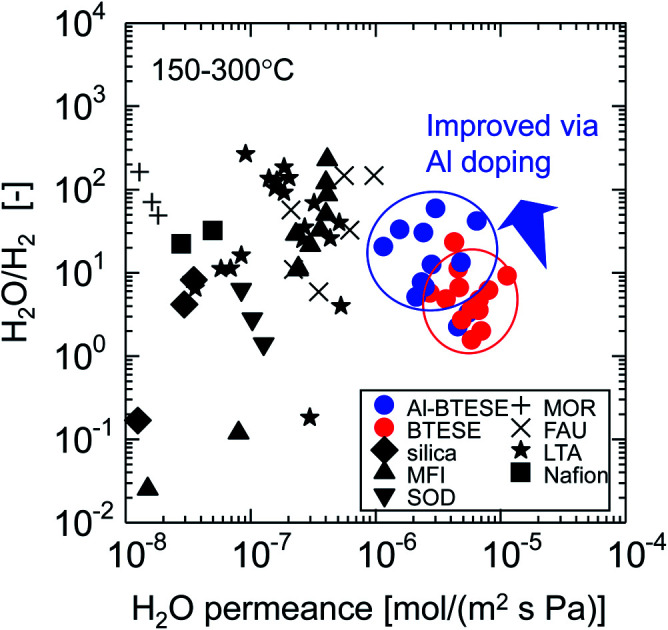

## 1. Introduction

Membrane technology provides advantages such as a low level of energy consumption, high-quality separation, and compactness. Membranes are being used for many applications such as desalination of sea water, concentration of fluids, wastewater treatment, and so on, most of which use organic polymeric membranes. In addition, the application of inorganic membranes is expected to extend the utility of membrane technology by providing high levels of thermal and chemical stability as well as excellent levels of permselectivity. An A-type zeolite membrane was the first commercialized inorganic version, and was used in the dehydration of alcohols. During ethanol dehydration, these membranes have shown a separation factor >10000 and a level of water permeance that reached more than 10^−6^ mol (m^−2^ s^−1^ Pa^−1^), which is superior to organic membranes.^1,2^ Conventional A-type membranes lack stability for use under high water content and acidic conditions, however, and other types of zeolite membranes such as T-type and CHA zeolites have been developed to overcome the problems associated with A-type zeolite membranes.^3–6^ At present, zeolite membranes such as LTA, MFI, CHA, FAU, MOR, and others have been widely studied and have shown promise for use in both liquid and gas-phase separations.^7^

Silica has also been extensively investigated for use in fabricating inorganic membranes due to its thermal stability above 1000 °C and a high level of chemical stability when exposed to various types of organic solvents. Silica membranes usually show hydrogen permeance that ranges from 10^−7^–10^−5^ mol (m^−2^ s^−1^ Pa^−1^) with a H_2_/N_2_ permeance ratio that can range from 100–10000, which makes them comparable to, or even higher than, zeolite versions.^8–11^ Such qualities have raised expectations for their use in various separation systems. The application of conventional silica membranes is limited, however, due to the lack of hydrothermal stability. Reportedly, water hydrolyzes the siloxane bonds to produce silanol groups even at room temperature, which is followed by recondensation of silanol groups to form siloxane bonds, which results in a densification of the silica membrane structure.^12–14^ The improvement of hydrothermal stability is one of the most important challenges for silica membranes and has been pursued by many researchers.^1,15–19^ Over the past two decades, the use of organosilica membranes prepared from an organically bridged alkoxysilane such as 1,2-bis(triethoxysilyl)ethane (BTESE) have dramatically improved the hydrothermal stability. Castricum *et al.* prepared an organosilica membrane derived from a mixture of BTESE and methyltriethoxysilane (MTES) and applied it to the pervaporation dehydration of an aqueous solution of *n*-butanol at 150 °C where the water content was 5 wt%.^20^ The membrane maintained high flux and high selectivity for almost two years, while conventional silica membranes lost their performance within a week. Those researchers explained that the improved hydrothermal stability was due to characteristics such as non-polarity, non-vulnerability to hydrolysis, and to the flexibility of organic linking units. We applied BTESE-derived organosilica membranes to reverse osmosis and reported stable performance at 90 °C even with a water content of almost 100%.^21^ Based on such results, organosilica membranes could be used in fields such as gas separation,^11,19,22–25^ dehydration of organic solvents,^26–29^ and water purification.^21,30^

Recently, we attempted the first use of BTESE-derived organosilica membranes in a humid-gas separation at 80–200 °C. In this study, we focused on the possibility of future applications such as in steam recovery from combustion flue gas streams and in dehydration reactions in membrane reactors.^31,32^ With the use of a hydrothermally stable intermediate layer, the BTESE-derived membrane maintained excellent water permeance of 5 × 10^−6^ mol (m^−2^ s^−1^ Pa^−1^) with a H_2_O/N_2_ permeance ratio that reached as high as 350 during steam permeation for 15 days at 200 °C under water vapor pressure of 200 kPa, which confirmed the strong potential of this technology for humid-gas separations.^33^ At present, the selectivity of organosilica membranes is due primarily to the contribution of molecular sieving where the permeation of larger molecules is more sterically hindered by the pore walls. In addition, we can expect the adsorption of water in the membrane pores to further block the permeation of non-condensable gases, which is well known to occur in microporous membranes with high selectivity. In other words, if organosilica membranes, which are reported to be moderately hydrophilic due to hydrophilic silanol groups and hydrophobic organic linking units in amorphous silica networks, can be tuned to be more hydrophilic, higher selectivity in humid-gas separation should be realized by both molecular sieving and blocking.

Metal doping into the silica membrane structure has shown the possibility to improve the affinity of silica and organosilica membranes with specific molecules. We found that Ag doping enhanced the affinity of a bis(triethoxysilyl)methane (BTESM)-derived membrane with propylene.^34^ The surface diffusion of propylene was enhanced, which resulted in improved selectivity of propylene over propane with results as high as 32.5. Qi *et al.* reported Nb doping into organosilica membranes derived from BTESE.^35,36^ Due to the improved Lewis acidity derived from the incorporation of a Nb dopant, the membrane fired at 450 °C showed surprisingly low CO_2_ permeance, and thus H_2_/CO_2_ selectivity was as high as 220. By considering these results, metal doping with an appropriate selection of metal type should improve the hydrophilicity of organosilica membranes and result in high selectivity in humid-gas separation.

In the present study, Al was selected as a candidate for the doping of BTESE-derived organosilica membranes for improved performance in steam recovery at high temperature. In the literature,^37^ doped Al atoms can be incorporated in the organosilica structure in a four- to six-fold configuration, which would lend a tetrahedral connection to the framework (Al(OSi)_4_) and an AlO_6_ octahedra configuration (Al^3+^ ions coordinated with SiOH groups and H_2_O), respectively. The incorporation of Al atoms could control the pore sizes and simultaneously improve the hydrophilicity of the organosilica structure that is widely accepted in zeolite chemistry.^38,39^ We prepared Al-doped organosilica membranes from BTESE with different Al/Si contents of 0.05–0.2, and evaluated their humid-gas separation performance under temperatures and water vapor pressures ranging from 80 to 200 °C and 10 kPa to 360 kPa, respectively. In addition, water adsorption and desorption experiments were conducted to elucidate the performance. As a result, we have confirmed the improved performance made *via* Al doping into organosilica membranes for use in humid-gas separation at moderate-to-high temperatures.

## 2. Experimental

### 2.1 Sol and powder preparation

1,2-Bis(triethoxysilyl)ethane (BTESE) was purchased from Gelest and used as a precursor without purification. BTESE- and Al-BTESE-derived polymeric sols were prepared following a previously reported procedure.^24,40^ For BTESE-derived sols, BTESE, water, and hydrochloric acid was mixed in an ethanol solvent. On the other hand, for Al-BTESE-derived sols, BTESE was mixed with aluminum nitrate (Al(NO_3_)_3_·9H_2_O), water and nitric acid in an ethanol solvent. Then, the solutions were stirred at 50 °C for 1 h, which promoted hydrolysis and condensation to obtain BTESE- and Al-BTESE-derived polymeric sols. The molar ratios of water (water molar ratio, WR) and acid catalyst over BTESE (acid molar ratio, AR) were controlled at 240 and 10^−2^–10^−1^, respectively, where the concentration of BTESE was 5 wt% in the initial composition. The amount of aluminum nitrate to be added was determined in order to maintain an Al/Si atomic ratio of 0–0.2. BTESE- and Al-BTESE-derived powders, respectively, were prepared by drying BTESE- and Al-BTESE-derived polymeric sols with an AR of 10^−1^ and calcination at 300 °C under a nitrogen flow.

### 2.2 Membrane fabrication

BTESE- and Al-BTESE-derived membranes, respectively, were fabricated by coating BTESE- and Al-BTESE-derived polymeric sols on an intermediate layer prepared from a BTESE-derived colloidal sol, as reported elsewhere.^33^ Briefly, an α-alumina porous tube (average pore size: 1 μm, outside diameter 10 mm, length 100 mm, Nikkato Cor., Japan) was used as a support. First, α-alumina particles (diameter of 1–2 μm and 0.2 μm) were tuned to a concentration of 5–10 wt% using BTESE-derived colloidal sols and were coated onto the outside of the tube to smooth the surface, which was followed by calcination at 300 °C. Then, a BTESE-derived colloidal sol diluted to 0.5 wt% by water was coated *via* a so-called hot-coating method, which was followed by calcination at 300 °C under a nitrogen flow. This procedure was repeated several times to form a continuous intermediate layer with an average pore size of around 1 nm. BTESE- and Al-BTESE-derived separation layers, respectively, were fabricated by hot-coating BTESE- and Al-BTESE-derived polymeric sols, both of which were diluted to 0.25 wt% by ethanol, followed by calcination at 300 °C under a nitrogen flow for 30 min. The cross-sectional SEM images of the BTESE- and Al-BTESE-derived membranes can be found in ESI-1 (ESI†). In the present study, BTESE- and Al-BTESE-derived membranes with different Al/Si ratios were prepared using sols, as summarized in Table 1.

**Table 1:** Membranes prepared from different types of sols

Membrane	Type	Al/Si [−]	AR [−]	WR [−]
M-0	BTESE	0	10^−1^	240
M-0.05	Al-BTESE	0.05	10^−1^	240
M-0.1	Al-BTESE	0.1	10^−1^	240
M-0.2	Al-BTESE	0.2	10^−1^	240
M-0*	BTESE	0	10^−2^	240

### 2.3 Characterization of sols and powders

The sol size was measured using a dynamic light scattering (DLS) analyzer (Malvern Zetasizer Nano ZS). X-ray diffraction (XRD), adsorption, and temperature programed desorption (TPD) were performed on powders using a D2 PHASER 2nd Gen. (Bruker Cor., Germany), a BELMAX (BELL Cor., Japan), and a BELCAT (BELL Cor., Japan), respectively. The powders were dried at 200 °C for longer than 12 h prior to use for characterization.

### 2.4 Permeation experiment

Single-gas permeation and binary humid-gas separation experiments were performed using an in-house fabricated apparatus described in our previous reports.^32,33^ For the single-gas permeation, seven types of single-gases (He, H_2_, CO_2_, N_2_, CH_4_, CF_4_, SF_6_) were fed to the outside of the tube-shaped membrane at 80–200 °C with a pressure of 200 kPa (abs), while the permeate stream was released at atmospheric pressure. The activation energy for permeation of the *i*-th component, *E*_P,*i*_ [J mol^−1^], was evaluated *via* the modified gas-translation model represented by eqn (1).^41^ The details can be found in ESI-2 (ESI†).1
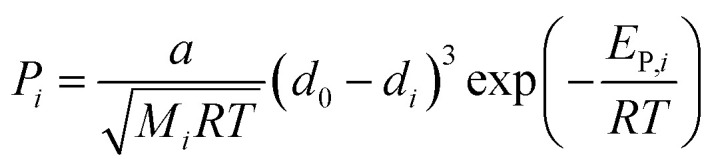


In eqn (1), *P*_*i*_ [mol (m^−2^ s^−1^ Pa^−1^)], *M*_*i*_ [g mol^−1^], *R* [J (mol^−1^ K^−1^)], *T* [K], *d*_0_ [nm], and *d*_*i*_ [nm] are the permeance, molecular weight, gas constant, temperature, pore size, and molecular size, respectively. In the equation, *a* [nm^−3^] is a constant that depends only on membrane structures such as pore size, thickness and porosity independent of the permeating gas species.

For the binary humid-gas separation, liquid water and single-gases (H_2_, N_2_) were mixed at a certain flow rate, and were then completely evaporated prior to being fed to the outside of the membrane. The pressure of the feed stream was controlled from 100 to 400 kPa (abs) at a temperature of 80–150 °C where the pressure of the permeate stream was controlled from 0 to 100 kPa (abs). The total flow rate of the feed stream was maintained at 10 L min^−1^ (STP). The permeance during binary humid-gas separation was calculated using eqn (2) under the assumption of a cocurrent flow.2
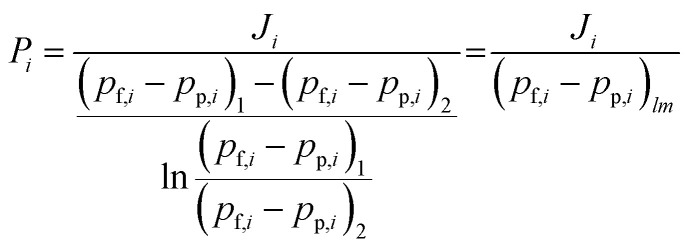


In eqn (2), *J*_*i*_ [mol (m^−2^ s^−1^)], *p*_f,*i*_ [Pa], and *p*_p,*i*_ [Pa] are the permeating flux and the partial pressures of the feed and the permeate streams of the *i*-th component, respectively. The subscripts 1 and 2 indicate the inlet and outlet of the membrane module, respectively. This equation is commonly used to calculate the permeance in a binary system, however, the applicability of its use is limited by conditions such as the flow rate of the feed and permeate. We proposed a guideline to confirm the applicability.^42^ In this study, eqn (2) was applied when the operation conditions followed the guidelines; otherwise, a numerical computation was performed on the experimental data to obtain the permeance in humid-gas separations.

## 3. Results and discussion

### 3.1 Effect of Al doping on sols, gels and membranes

**Fig. 1:**
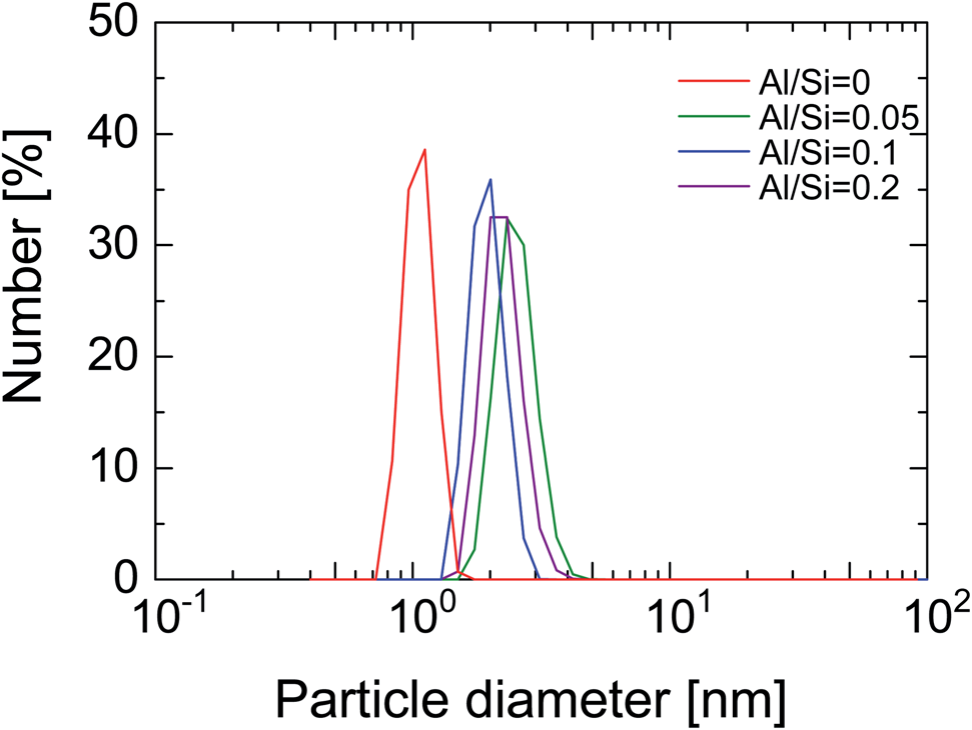
Particle size distribution of BTESE-acid (Al/Si = 0) and Al-BTESE (Al/Si = 0.05–0.2) sols measured by DLS.

Fig. 1 shows the size distribution of BTESE-acid and Al-BTESE sols with different Al/Si ratios. The sols showed a monomodal size distribution centered around 1–2 nm, all of which would be suitable to prepare a separation top layer on the intermediate layer with average pore sizes of around 1 nm.^33^ In detail, the sizes of the BTESE-acid sols were approximately 1 nm, while those of Al-BTESE sols were around 2 nm independent of the Al/Si ratios. Al doping, which reportedly can be incorporated into organosilica networks as Al^3+^ ions at low temperature^40^ slightly increased the sol size, while Zr doping significantly increased the size due to the formation of a Si–O–Zr bond between the original organosilica particles, as reported elsewhere.^43^ It should be noted that all prepared sols were clear, however, large particles with a micrometer-order appeared in the sol of Al/Si = 0.2 when cooled to room temperature, which suggested a phase separation/aggregation of excess Al incorporated in the organosilica network of this class. Therefore, Al/Si ratios higher than 0.2 were not examined in the present study.

**Fig. 2:**
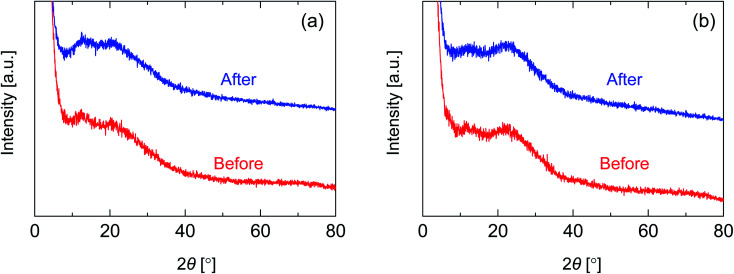
XRD patterns for BTESE- (a) and Al-BTESE-derived (Al/Si = 0.1) powders (b) before and after hydrothermal treatment (200 °C, water vapor pressure: 200 kPa (abs), 24 h).

In order to investigate the structure of organosilica networks derived from BTESE- and Al-BTESE-derived sols, BTESE- and Al-BTESE-derived powders were prepared *via* firing at 300 °C under a N_2_ flow similar to the membrane fabrication process. The XRD patterns of powders prepared from BTESE- (a) and Al-BTESE-derived (Al/Si = 0.1) sols (b) can be found in Fig. 2. Additional XRD patterns of powders with Al/Si = 0.05 and 0.2 are reported in ESI-3 (ESI†). In Fig. 2, since the membranes were further evaluated under hydrothermal conditions, we included the XRD patterns before and after hydrothermal treatment at 200 °C under a water vapor pressure of 200 kPa (abs) for 24 h. Before the hydrothermal treatment, both BTESE- and Al-BTESE-derived powders showed broad peaks centered at 12 and 20, and 12 and 22°, respectively, which was assigned to the amorphous structure that typically originates from the Si–Si distance.^44^ The slight peak shift to a high 2*θ via* Al doping was the result of densification of the amorphous structure, as confirmed later. More importantly, sharp peaks were not observed for either of the powders, suggesting that Al atoms were incorporated into the amorphous silica structure with no crystallization. In addition, for both BTESE- and Al-BTESE-derived powders, the XRD patterns measured after hydrothermal treatment were almost the same as those before hydrothermal treatment. This suggests that both BTESE- and Al-BTESE-derived gels maintained amorphous structures that showed neither crystallization nor densification under hydrothermal conditions.

**Fig. 3:**
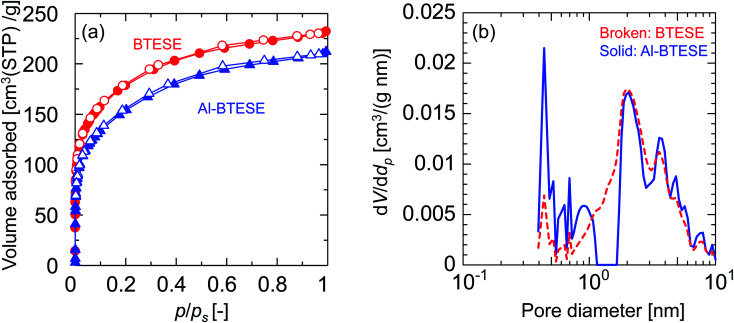
Nitrogen sorption properties for BTESE- and Al-BTESE-derived (Al/Si = 0.1) powders. (a) Adsorption and desorption isotherms at −196 °C. Closed and open symbols indicate adsorption and desorption, respectively. The results of Al/Si = 0.05 and 0.2 can be found in ESI-4 (ESI†). (b) Pore diameter distribution calculated *via* NLDFT.

Fig. 3(a) shows the nitrogen adsorption and desorption isotherms at −196 °C for BTESE- and Al-BTESE-derived (Al/Si = 0.1) powders. Both powders showed a large amount of adsorption even at low *p*/*p*_s_ and achieved adsorption greater than 200 cm^3^(STP) per g at *p*/*p*_s_ that approximated 1. This confirmed the microporous structures and high porosity of these powders. In addition, the volume adsorbed by the Al-BTESE-derived powder, which was somewhat smaller than that of BTESE-derived version, suggested a densification of the pore structure *via* Al doping. To confirm this, the pore size distribution of each powder was analyzed *via* NLDFT, as shown in Fig. 3(b). The pores smaller and larger than 1.2 nm were assigned as inner-particle and inter-particle pores, respectively, where they respectively corresponded to pores formed in siloxane networks and those formed as spaces between the sol particles. In a range of pore diameters from 1.2–10 nm, BTESE- and Al-BTESE-derived powders showed a similar pore size distribution, probably due to the similar size of BTESE-acid and Al-BTESE sols as confirmed in Fig. 1. On the other hand, in the range of 0.4–1.2 nm, Al-BTESE-derived powder showed larger values for d*V*/d*d_p_* than those of the BTESE-derived version. In particular, this was more significant for the extremely smaller sized pores of 0.4–0.6 nm, which were effective for molecular separation as a bottleneck, and confirmed the densified pore structure of the Al-BTESE-derived powder.

**Fig. 4:**
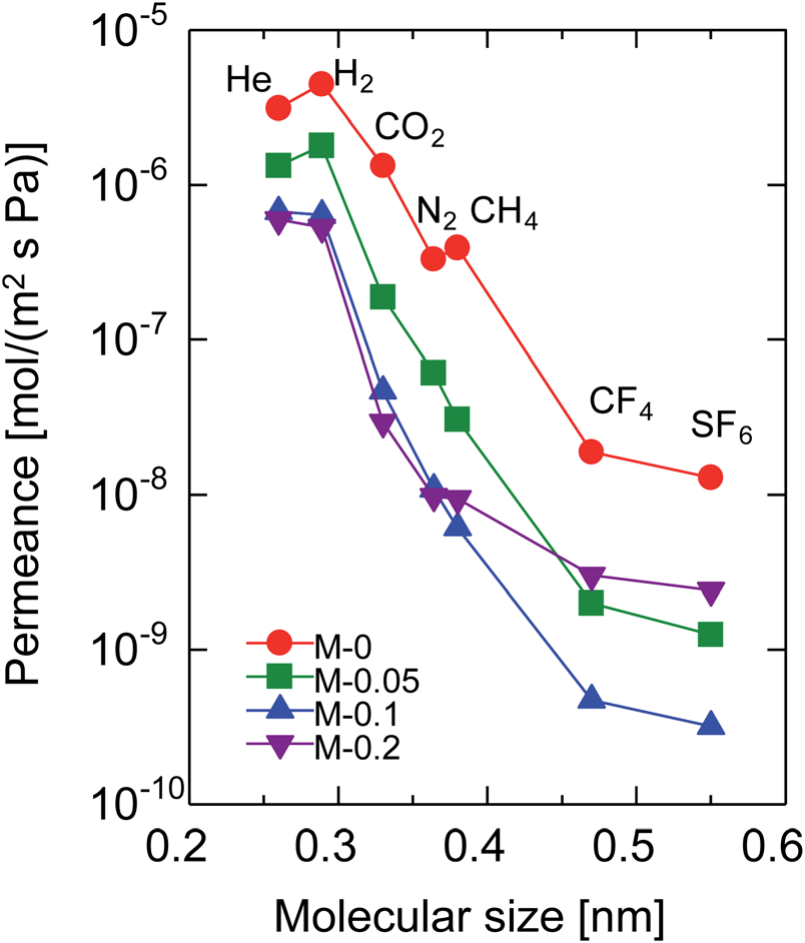
Single-gas permeance through BTESE- and Al-BTESE-derived membranes at 200 °C as a function of molecular size.

**Fig. 5:**
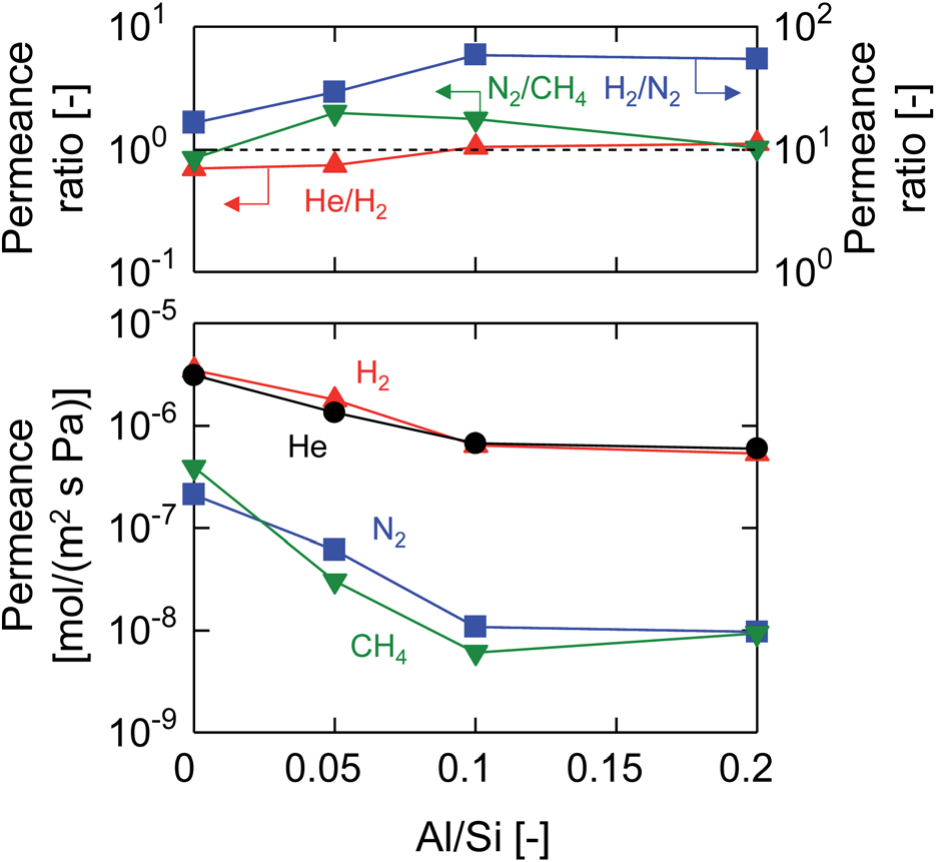
Single-gas permeance and permeance ratios at 200 °C as a function of the Al/Si ratio (M-0, M-0.05, M-0.1, and M-0.2).

Fig. 4 shows the molecular size dependency of single-gas permeance through M-0, 0.05, 0.1 and 0.2 at 200 °C. It should be noted that these membranes were prepared with the same AR of 10^−1^ while the Al/Si ratios differed from 0 to 0.2, as summarized in Table 1. The membranes showed a level of helium permeance that reached a level as high as 0.6–3.1 × 10^−6^ mol (m^−2^ s^−1^ Pa^−1^), and confirmed the high permeability of the membranes. The permeance of the membranes decreased as the molecular size increased, which confirmed that the molecular sieving originated from a subnanoporous structure that dominated the single-gas permeation properties. Fig. 5 summarizes the effect of the Al/Si ratio on the permeance and on the permeance ratios. As the Al/Si ratio increased, values for the permeance of helium, hydrogen and nitrogen gradually decreased. The decrease was more significant for nitrogen than it for hydrogen, and resulted in a gradual increase in the H_2_/N_2_ permeance ratio of from 17 (Al/Si = 0) to 55 (Al/Si = 0.2). This clearly indicates that Al doping densified the pore structure of BTESE-derived membranes, which was confirmed *via* the nitrogen adsorption of the powders (Fig. 3). The activation energies for hydrogen and nitrogen permeation, which tended to increase from 2.2 to 8.9 kJ mol^−1^ and from 1.8 to 4.0 kJ mol^−1^, respectively, as listed in Table S-1 (ESI-2, ESI†), also confirmed the densification of the amorphous organosilica networks. More importantly, the He/H_2_ permeance ratio of 0.70 (Al/Si = 0), which approximated that of the Knudsen selectivity of 0.71 where selectivity is dominated by Knudsen diffusivity and was obtained as a ratio of the square root of the molecular weight, was improved to 1.1 (Al/Si = 0.2). In addition, the N_2_/CH_4_ permeance ratio of 0.84 (Al/Si = 0), which was slightly larger than the Knudsen selectivity of 0.76, was also increased to 2.0 (Al/Si = 0.05). This observation in permselective properties, that is, change from hydrogen- and methane-selectivity to helium- and nitrogen-selectivity, clearly indicated that the permeation mechanism changed from a Knudsen flow to a molecular sieving flow, again confirming the densification of the pore structure *via* Al doping. These results showed good agreement with those in the literature for attempted Al doping into bis(triethoxysilyl)methane (BTESM)-derived membranes.^37^ Finally, the permeance analysis using the m-GT model was conducted on M-0, M-0.05, M-0.1, and M-0.2 and confirmed that the average pore size of the membranes decreased from 0.56 to 0.42 nm as the Al/Si ratio was increased from 0 to 0.2 (ESI-5, ESI†). It should be noted that methane permeance slightly increased when Al/Si was increased from 0.1 to 0.2, which resulted in a slightly decreased permeance ratio of N_2_/CH_4_. This suggests that non-negligible numbers of large pores such as pinholes were generated in the membrane structure at Al/Si = 0.2, which is schematically shown in ESI-6 (ESI†), probably by the micrometer-ordered particles in Al/Si = 0.2 sol that formed due to the saturation of Al in the BTESE-derived structure.

### 3.2 Humid-gas separation performance

**Fig. 6:**
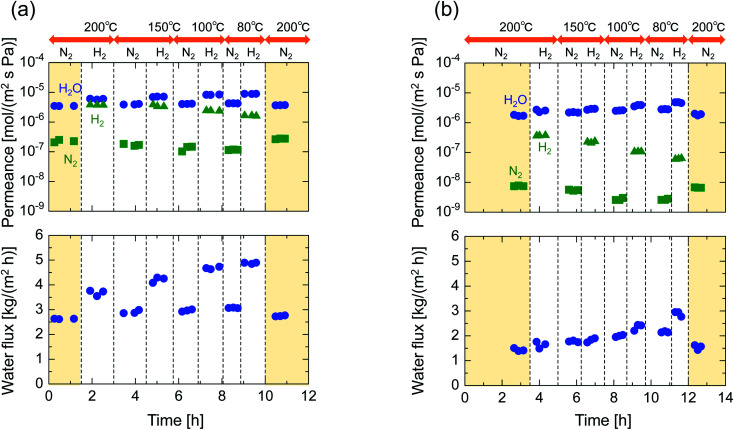
Time courses of binary humid-gas separation performances for (a) M-0 and (b) M-0.1 under a constant water mole fraction, *x*_w_, of 0.1 at 80–200 °C. Feed and permeate pressure were maintained at 100–130 kPa (abs) and ∼0 kPa (abs), respectively. Nitrogen and hydrogen were fed alternately as a non-condensable gas for each temperature while water vapor was fed continuously.

**Fig. 7:**
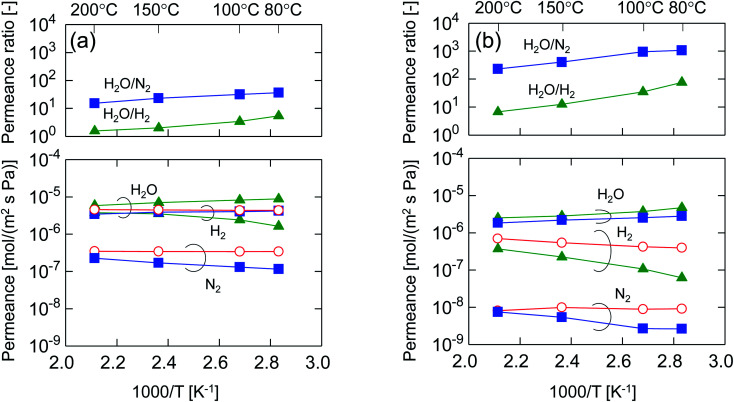
Temperature dependency of permeance and permeance ratios through (a) M-0 and (b) M-0.1 in humid-gas separation and single-gas permeation. For humid-gas separation, the water mole fraction of the feed stream, *x*_w_, was maintained at 0.1. Feed and permeate pressures were maintained at 100–130 kPa (abs) and ∼0 kPa (abs), respectively (circle: single-gas; triangle: H_2_O/H_2_; square: H_2_O/N_2_).

After single-gas permeation experiments, the membranes were used for binary humid-gas separation. Fig. 6 shows the time course of permeance and the water flux values for (a) M-0 and (b) M-0.1 during humid-gas separation experiments. The experiment was operated with a constant water mole fraction in the feed, *x*_w_, of 0.1 at temperatures ranging from 80–200 °C with feed and permeate pressure maintained at 100–130 kPa and ∼0 kPa, respectively. This system approximately corresponds to steam recovery from combustion flue gas. It is noteworthy that nitrogen and hydrogen were fed alternately as non-condensable gases for each temperature while water vapor was fed continuously. Both membranes showed water permeance as large as ∼several 10^−6^ mol (m^−2^ s^−1^ Pa^−1^). It is interesting that this value for permeance is larger than that of hydrogen, although the molecular size of water (0.2955 nm (ref. 45)) is larger than that of hydrogen (0.289 nm (ref. 46)). In addition, the water flux of 1.5–4.7 kg (m^−2^ h^−1^) was also large even though the feed stream contained only 10 mol% of water vapor at the inlet. H_2_O/N_2_ separations at 200 °C were carried out for both the initial and the last experiment with a sequential procedure and showed approximately the same performance, confirming the hydrothermal stability for both types of BTESE- and Al-BTESE-derived membranes as well as the reproducibility of these experiments. The performances (permeance and permeance ratios) are summarized as a function of temperature in Fig. 7. As the temperature was decreased from 200 to 80 °C, water permeance increased from 3.4–5.9 × 10^−6^ to 4.2–8.8 × 10^−6^ mol (m^−2^ s^−1^ Pa^−1^) for M-0 and from 1.8–2.5 × 10^−6^ to 2.8–4.7 × 10^−6^ mol (m^−2^ s^−1^ Pa^−1^) for M-0.1, which suggests the water molecules underwent surface diffusion. On the other hand, the permeance of non-condensable gases (H_2_ and N_2_) in single and mixture systems tended to decrease as the temperature decreased. Since the decrease was more dramatic in humid-gas separation than in single-gas permeation for both hydrogen and nitrogen and for both membranes, it is plausible to infer that adsorbed water in the membrane pores prevented the permeation of non-condensable gases, which is often reported as the “blocking effect”.^31,47^ As a result, the permeance values for both H_2_O/H_2_ and H_2_O/N_2_ were significantly improved at low temperature. At 80 °C, M-0 reached H_2_O/H_2_ = 5.4 and H_2_O/N_2_ = 37, while M-0.1 reached H_2_O/H_2_ = 76 and H_2_O/N_2_ = 1100 for M-0.1. It is important to note that under all experimental conditions, M-0.1 recorded much larger permeance ratios for H_2_O/H_2_ and H_2_O/N_2_ compared with that of M-0, which confirms that Al doping into organosilica membranes can improve selectivity in humid-gas separations.

**Fig. 8:**
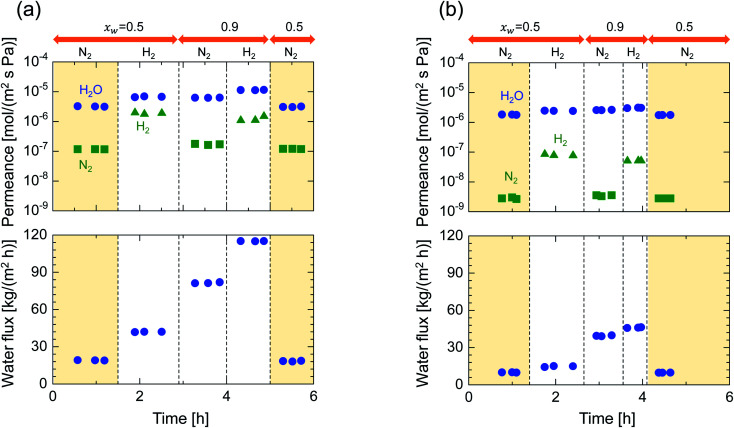
Time courses for the binary humid-gas separation performances of M-0 (a) and M-0.1 (b) under *x*_w_ values of 0.5 and 0.9 at a constant temperature of 150 °C. Feed and permeate pressure were maintained at 400 kPa (abs) and atmospheric pressure, respectively. Nitrogen and hydrogen were fed alternately as a non-condensable gas for each value of *x*_w_ while the water vapor was fed continuously.

**Fig. 9:**
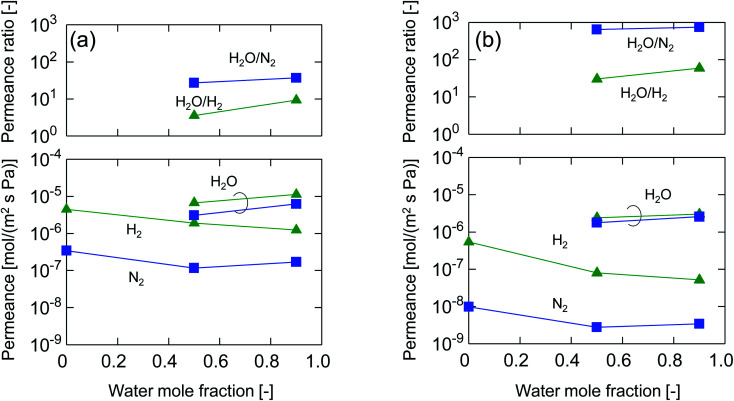
Water mole fraction, *x*_w_, dependency on the permeance and permeance ratios through M-0 (a) and M-0.1 (b) during humid-gas separation and single-gas permeation at 150 °C, where the feed and permeate pressures were maintained at 400 kPa (abs) and atmospheric pressure, respectively (triangle: H_2_O/H_2_; square: H_2_O/N_2_).

In order to examine the use of BTESE-derived and Al-BTESE-derived membranes under high vapor pressure, humid-gas separation was further investigated under a pressurized feed stream. Fig. 8 shows the time courses of binary humid-gas separation performances for (a) M-0 and (b) M-0.1 at a constant temperature of 150 °C. During this experiment, the total pressures of the feed and permeate streams were maintained at 400 kPa (abs) and atmospheric pressure, respectively, where *x*_w_ was controlled at 0.5–0.9, which corresponds to a water vapor pressure in the feed of 200–360 kPa (abs). This system approximately corresponds to steam recovery from the type of high-pressure stream that often is observed in continuous chemical processes, and this high water vapor pressure in both the feed and permeate stream was more severe to membranes than the example of low vapor pressure shown in Fig. 6. After changing the operation conditions, the permeance and water flux immediately responded in ten minutes, and had shown a stable value under each condition. Both membranes showed water permeance as large as ∼several 10^−6^ mol (m^−2^ s^−1^ Pa^−1^), which is similar to that recorded in Fig. 6. However, water flux, which is more important from a practical viewpoint, reached at most 115 kg (m^−2^ h^−1^) for M-0 and 45 kg (m^−2^ h^−1^) for M-0.1. Those values are significantly larger than the case shown in Fig. 6. In addition, the mixture separation of H_2_O/N_2_ with an *x*_w_ of 0.5 maintained a stable performance at the initial and the last measurement of the sequential experiment for both membranes. This again confirmed the hydrothermal stability of BTESE-derived and Al-BTESE-derived membranes even under a water-vapor pressure that reached as high as 200–360 kPa (abs). We previously reported stability for as long as 15 days for a BTESE-derived membrane.^33^ The present study further confirmed the stability of an Al-BTESE-derived membrane. Fig. 9 summarizes the humid-gas separation performance as a function of the *x*_w_. Hydrogen and nitrogen permeance with a *x*_w_ = 0 corresponds to the single-gas permeance at 150 °C. Under all experimental conditions, M-0.1 showed larger permeance ratios for H_2_O/H_2_ and H_2_O/N_2_ compared with those of M-0, and both H_2_O/H_2_ and H_2_O/N_2_ reached the maximum permeance ratios of 59 and 740, respectively, which is similar to the results shown in Fig. 7. As the *x*_w_ increased, the hydrogen and nitrogen permeance decreased while water permeance did not, suggesting a blockage of the permeation of non-condensable molecules combined with adsorbed water. Thus, the permeance ratio improved with a larger *x*_w_. It is noteworthy that the decrease in hydrogen and nitrogen permeance was more significant in M-0.1 than in M-0, which implies that the ability to block non-condensable gases can be enhanced *via* Al doping. This will be discussed in the following section.

**Fig. 10:**
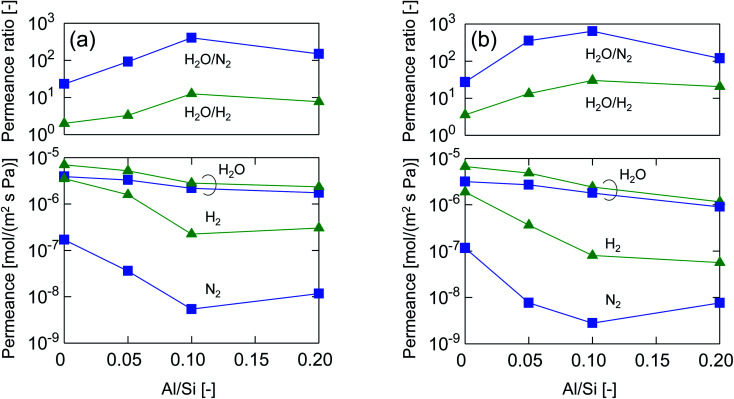
Permeance and permeance ratios in binary humid-gas separation at 150 °C as a function of the Al/Si ratio (M-0, M-0.05, M-0.1 and M-0.2) (*x*_w_: 0.1, feed: 100–130 kPa (abs), permeate: ∼0 kPa (abs) (a), *x*_w_: 0.5, feed: 400 kPa (abs), permeate: atmospheric pressure (b)).

Fig. 10 summarizes the permeance and permeance ratios as a function of the Al/Si ratio under a *x*_w_ of 0.1 where the values for feed and permeate pressures were 100–130 kPa (abs) and ∼0 kPa (abs), respectively (a), and under a *x*_w_ of 0.5 where values for the feed and permeate pressures were 400 kPa (abs) and atmospheric pressure, respectively (b). For both figures, as a general trend, water permeance was slightly decreased but hydrogen and nitrogen permeance was dramatically decreased following an increase in the Al/Si ratio, which dramatically improved the permeance ratios. This clearly indicates the effectiveness of Al doping to improve selectivity in humid-gas separations. In addition, this trend was similar to that in single-gas permeation where the permeance ratios of He/H_2_ and H_2_/N_2_ increased with an increase in Al/Si, as shown in Fig. 5. At an Al/Si ratio of 0.2, the permeance ratio of H_2_O/N_2_ was decreased probably due to large pores such as pinholes, and this was similar to the permeance ratio of H_2_/SF_6_ during single-gas permeation, as shown in ESI-7 (ESI†). Based on these similarities of permeance ratios for humid-gas separation and single-gas permeation, it can be reasonably concluded that molecular sieving, which dominates single-gas permeation, was one of the dominant factors in humid-gas separation. In detail, by doping Al with an Al/Si ratio of 0.1, H_2_/N_2_ and H_2_/SF_6_ in single-gas permeation (Fig. 5 and ESI-7 (ESI†)), which is dominated by molecular sieving, increased from 16 to 59 and from 600 to 2000, respectively, which represents an increase of from 3.3- to 3.6-fold. On the other hand, H_2_O/H_2_ and H_2_O/N_2_ in humid-gas separation (Fig. 10(b)) increased from 3.6 to 30 and from 27 to 640, respectively, which represents an increase of from 8.5- to 24-fold. Since Al doping contributed to the improvement in permeance ratios in humid-gas separation more significantly than in single-gas permeation, it is suggested that the increased permeance ratios in humid-gas separation were not only improved by molecular sieving but also by additional effects that included an improved blockage of permeation by non-condensable gases.

### 3.3 Effect of Al doping on improved performance

**Fig. 11:**
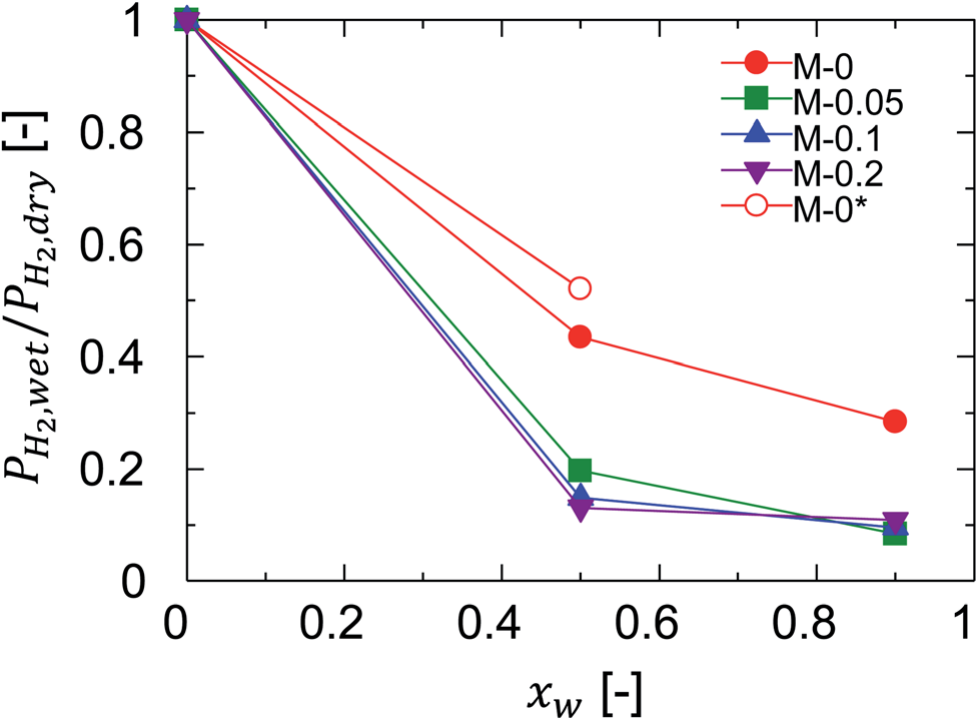
Relationship between *P*_H2,wet_/*P*_H2,dry_ and *x*_w_ at 150 °C where the feed and permeate pressures were maintained at 400 kPa (abs) and atmospheric levels, respectively. M-0, M-0.05, M-0.1, and M-0.2 were prepared with an AR = 10^−1^ while M-0* was prepared with an AR = 10^−2^.

The previous section revealed the effectiveness of Al doing to improve the permeance ratio in humid-gas separation, and suggested contributions to effects such as improvement in the blockage of permeation of non-condensable gases. For clarification, the blocking effect was quantified as the ratio of hydrogen permeance in humid-gas separation over that in single-gas permeation, *P*_H2,wet_/*P*_H2,dry_, since hydrogen permeance is determined by network pores and is not affected by a small number of pinholes. *P*_H2,wet_/*P*_H2,dry_ ratios of one and zero correspond to a negligible and a complete blocking of hydrogen permeation by water vapor, respectively. Fig. 11 shows the relationship between *P*_H2,wet_/*P*_H2,dry_ and *x*_w_ at 150 °C where the feed and permeate pressures were maintained at 400 kPa (abs) and atmospheric levels, respectively. As a general trend, *P*_H2,wet_/*P*_H2,dry_ was decreased with an increase in *x*_w_, confirming the blocking effect where a larger amount of water molecules was adsorbed by pores to block the permeation of non-condensable gases such as hydrogen with a higher *x*_w_. Remarkably, the ratios of *P*_H2,wet_/*P*_H2,dry_ for M-0.05, M-0.1, and M-0.2 (Al-BTESE-derived) were significantly smaller than that of M-0 (BTESE-derived), which corresponds to an improved blocking effect for Al-BTESE-derived membranes. Therefore, it can be concluded that improvement in the permeance ratios of Al-BTESE-derived membranes during humid-gas separation (Fig. 10) were the result of the improvement in the blockage of non-condensable gas permeation as well as to molecular sieving. In addition, the ratios for *P*_H2,wet_/*P*_H2,dry_ of M-0.05, M-0.1, and M-0.2 were similar and independent of their different Al/Si ratios, which suggests that the small Al/Si ratio of 0.05 was enough to block hydrogen permeation *via* adsorbed water. There are two possible reasons that Al doping promoted water adsorption to improve the blocking of non-condensable gas permeation. First, water molecules were more easily adsorbed in the densified pores of the Al-BTESE-derived membranes; and second, Al doing improved the hydrophilicity of the membrane, which promoted water adsorption. For the sake of further discussion on this point, M-0* was included in Fig. 11. The pore size of M-0* was controlled *via* a previously reported technique by adjusting the acid molar ratio, AR, at 10^−2^ during sol preparation and no additives were added to the original BTESE-derived membranes.^22,24^ The H_2_/N_2_ single-gas permeance ratio was 42 for M-0* (ESI-8, ESI†), which was at the level of Al-BTESE-derived membranes (M-0.05, M-0.1 and M-0.2) in a range of from 29 to 59 (Fig. 5). That comparison indicated that the pore size of M-0* was comparable to those of M-0.05, M-0.1, and M-0.2. However, as shown in Fig. 11, the ratios of *P*_H2,wet_/*P*_H2,dry_ for both M-0* and M-0 were significantly larger than those for the Al-BTESE-derived membranes (M-0.05, M-0.1, and M-0.2). In addition, as illustrated in Fig. S-10 (ESI-8, ESI†), the relationship between *P*_H2,wet_/*P*_H2,dry_ and the permeance ratio of H_2_/N_2_ showed a *P*_H2,wet_/*P*_H2,dry_ value for the Al-BTESE-derived membranes that was significantly smaller than those of the BTESE-derived versions. That result shows that Al doping improved the hydrophilicity of the membrane and thus improved the permeance ratio during humid-gas separation.

**Fig. 12:**
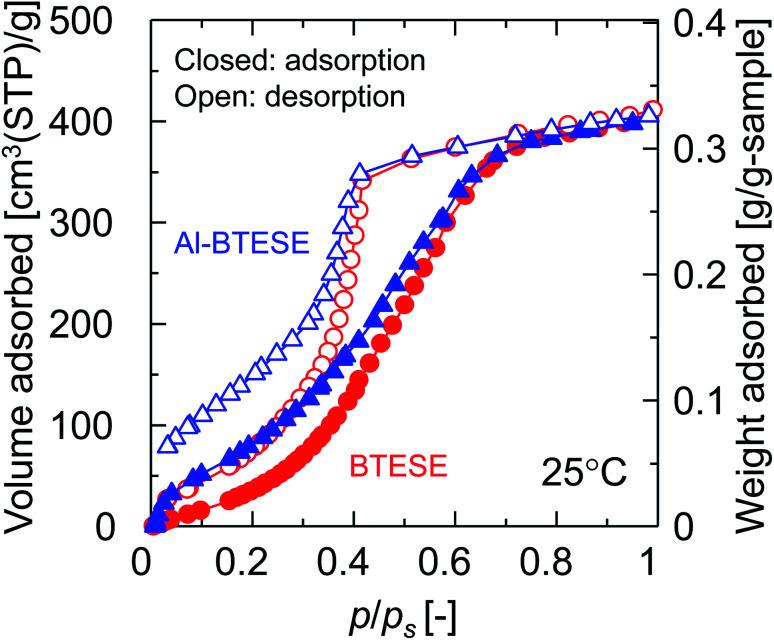
Water adsorption and desorption isotherms for BTESE- and Al-BTESE-derived (Al/Si = 0.1) powders at 25 °C. The circle/triangle and closed/open symbols indicate BTESE-/Al-BTESE-derived powders and the levels of adsorption and desorption, respectively. The results of Al/Si = 0.05 and 0.2 can be found in ESI-4 (ESI†).

Fig. 12 shows the water adsorption and desorption isotherms for BTESE- and Al-BTESE-derived (Al/Si = 0.1) powders at 25 °C. Both powders showed volumes of adsorption that could be as high as 400 cm^3^(STP) per g at values for *p*/*p*_s_ that approximated 1. For *p*/*p*_s_ < 0.7, the adsorbed volumes were larger for Al-BTESE-derived powders than for the BTESE-derived versions. That result was opposite that of nitrogen adsorption (Fig. 3(a)) where BTESE-derived powder showed a larger volume of adsorption compared with that of the Al-BTESE-derived version. These results confirmed that the structures of Al-BTESE-derived gels were more hydrophilic than the BTESE-derived versions. This is consistent with the water contact angles on BTESE- and Al-BTESE-derived films, which decreased from 74 to 52° as the Al/Si ratio increased from 0 to 0.2 (ESI-9, ESI†). In addition, the Al-BTESE-derived powder showed hysteresis that was much more significant than that of the BTESE-derived version at extremely low levels of *p*/*p*_s_. This indicates that a significant amount of water molecules were adsorbed even at extremely low levels of *p*/*p*_s_, again confirming the hydrophilicity of Al-BTESE-derived gels.

**Fig. 13:**
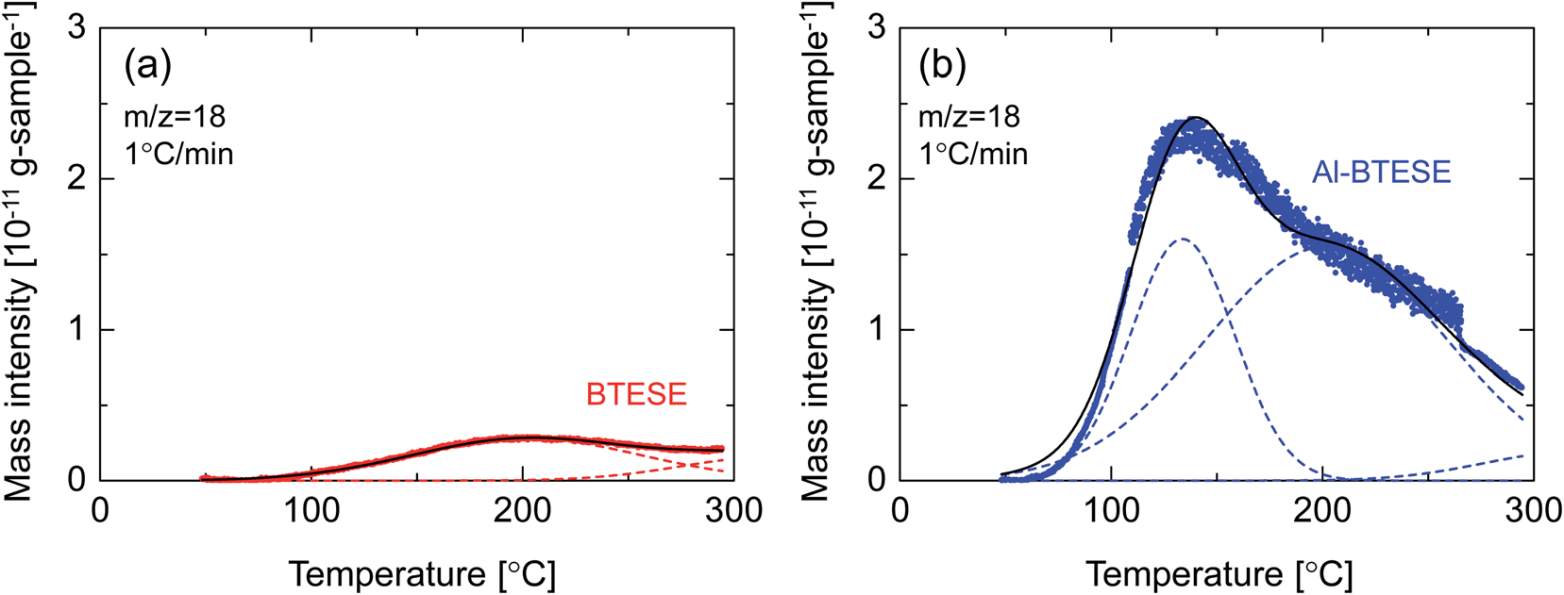
Water-temperature programmed desorption (H_2_O-TPD) curves of BTESE- (a) and Al-BTESE-derived (Al/Si = 0.1) (b) powders. The ramping rate was controlled at 1 °C min^−1^. Prior to the experiments, the powders were pre-adsorbed with water vapor at 50 °C under water vapor pressure of 5.6 kPa (saturated water vapor at 35 °C), and then dried under a dry helium flow at 50 °C for 5 h. The symbols and broken and solid curves indicate experimental results, deconvoluted peaks under the assumption of a Gaussian distribution and the total of these deconvoluted peaks, respectively.

**Fig. 14:**
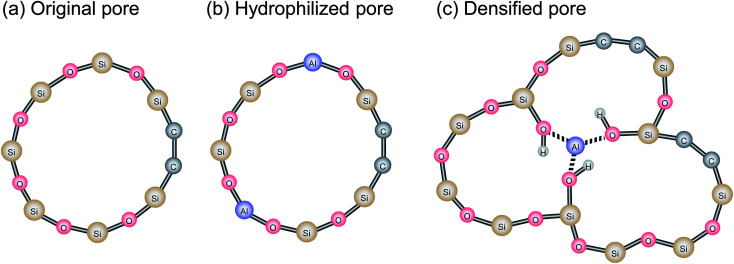
Network pore structures of Al-BTESE-derived gels.

Fig. 13 shows the water temperature programmed desorption (H_2_O-TPD) curves of BTESE- (a) and Al-BTESE-derived (Al/Si = 0.1) (b) powders. The powders were pre-adsorbed with water vapor at 50 °C under a water vapor pressure of 5.6 kPa (saturated water vapor at 35 °C), and then dried under a dry helium flow at 50 °C for 5 h, followed by temperature increases from 50 to 300 °C at 1 °C min^−1^. The time courses of mass signals and temperatures are shown in ESI-10 (ESI†). Obviously, the Al-BTESE-derived powder showed a mass signal (*m*/*z* = 18) that was much stronger than the BTESE-derived version at all temperatures, which indicated a larger amount of pre-adsorbed water remained after drying at 50 °C. The calibration curve showing the intensity (*m*/*z* = 18) and concentration of water vapor in the TPD experiment revealed that water had desorbed at rates of 2.2 and 16.1 mg per g-sample for BTESE-derived and Al-BTESE-derived powders, respectively. This result agreed well with the results shown in Fig. 12 with the Al-BTESE-derived powder showing significant hysteresis at low levels of *p*/*p*_s_. The TPD curve of BTESE-derived powders can be deconvoluted into two Gaussian distributions centered around 200 °C and higher than 300 °C. These distributions can be assigned to the water desorbed from small pores and to the dehydration of silanol groups (2SiOH → SiOSi + H_2_O), respectively. On the other hand, Al-BTESE-derived powder showed a TPD signal which must be deconvoluted into three Gaussian distributions centered at temperatures of approximately 130 °C as well as around 200 °C and higher than 300 °C. The distribution centered around 200 °C was larger for Al-BTESE-derived powder than for the BTESE-derived version due to the densified pore structure, as confirmed in Section 3.1. In addition, the desorption at approximately 130 °C could be considered as the result of water desorption from pores that are not so small in size but hydrophilized due to the presence of doped Al. According to the characterization *via*^27^Al MAS NMR (ESI-11, ESI†), Al atoms can be incorporated into an organosilica network structure as a tetrahedrally coordinated framework (Al(OSi)_4_) and as Al ions coordinated with silanol groups and water. Therefore, Al-BTESE-derived gels should have at least three types of pores, as follows. The first are pores derived from the original organosilica network with a traditional size distribution in principle (Fig. 14(a)). Considering the TPD results of BTESE-derived powders that show a small amount of desorption, most of the adsorbed water in these pores will be removed during drying at 50 °C before TPD measurement. Water pre-adsorbed by small original pores show peaks at approximately 200 °C, but the number of such pores is limited. The second type involves pores where Al atoms are tetrahedrally connected (Fig. 14(b)). In zeolite chemistry, cations associated with tetrahedrally coordinated aluminum are known to provide a specific interaction between water molecules and gels.^38^ Thus, these types of pores should be hydrophilized, which allow water molecules to abide in it during drying at 50 °C, even if the pore size is not so small. This then results in water desorption at approximately 130 °C. The third type involves pores where Al atoms are ionically linked to silanol groups (Fig. 14(c)). This ionic linking densifies the pore structure and produces a large number of small pores, as confirmed in Section 3.1, resulting in a large amount of water desorption around 200 °C.

**Fig. 15:**
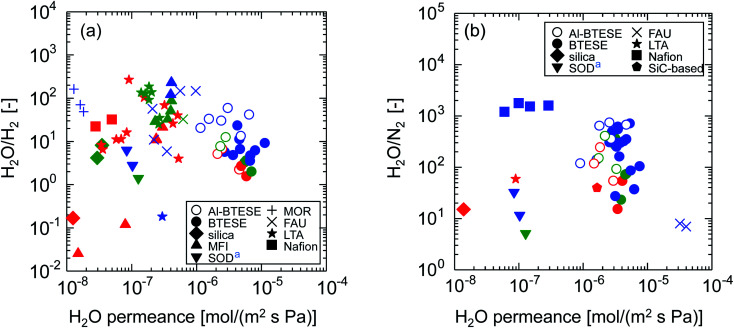
Relationships between permeance ratios ((a) H_2_O/H_2_, (b) H_2_O/N_2_) and water permeance at 150–300 °C.^32,33,47–61^ The value of each plot can be found in Table S-2 (ESI-12, ESI†). Symbols with different colors indicate different ranges of relative pressures, *p*/*p*_s_ (red: < 0.01, green: 0.01–0.05, blue: > 0.05). ^a^Performance in single-gas permeation.

Fig. 15 shows the relationships between permeance ratio ((a) H_2_O/H_2_, (b) H_2_O/N_2_) and water permeance at 150–300 °C. Symbols with different colors indicate different ranges of relative pressures, *p*/*p*_s_ (red: < 0.01, green: 0.01–0.05, blue: > 0.05). Water permeance and permeance ratios as a function of *p*/*p*_s_ can be found in ESI-12 (ESI†). High permeance ratios were achieved at higher *p*/*p*_s_ where membranes tended to take advantage of water adsorption to block non-condensable gas permeation. On the other hand, water permeance is apparently almost independent of *p*/*p*_s_ but strongly depends on membrane materials. In Fig. 15(a), Nafion and MOR zeolite membranes showed high H_2_O/H_2_ ratios of several tens even at low *p*/*p*_s_ with water permeance that was usually lower than 10^−7^ mol (m^−2^ s^−1^ Pa^−1^). MFI, FAU and LTA zeolite membranes showed somewhat improved water permeance of several 10^−7^ mol (m^−2^ s^−1^ Pa^−1^) with high H_2_O/H_2_ ratios of several tens. Among them, undoped BTESE-derived membranes showed excellent water permeance of several 10^−6^ mol (m^−2^ s^−1^ Pa^−1^) with moderate H_2_O/H_2_ ratios, which was usually in the range of several to ten. Remarkably, Al-BTESE-derived membranes achieved high H_2_O/H_2_ ratios of several tens, which clearly showed the improved selectivity *via* Al doping. Although water permeance was somewhat reduced with an improved H_2_O/H_2_ ratio, Al-BTESE-derived membranes still retained a water permeance of several 10^−6^ mol (m^−2^ s^−1^ Pa^−1^), which was much larger than those of other types of membranes. In addition, Fig. 15(b) demonstrates that Al doping improved the H_2_O/N_2_ ratios of BTESE-derived membranes with a slight expense of water permeance. Al-BTESE-derived membranes showed high-performance water permeance of several 10^−6^ mol (m^−2^ s^−1^ Pa^−1^) and H_2_O/N_2_ ratios of several hundreds based on the trade-off. Therefore, we concluded that the strategy of Al doping effectively achieves good humid-gas separation performance.

## 4. Conclusions

We attempted to improve the humid-gas separation performance of BTESE-derived membranes *via* Al doping. In the present study, two effects of Al doping on gel structure were confirmed. The first effect involved a densification of the pore structure. In the single-gas permeation of BTESE- and Al-BTESE-derived membranes with different Al/Si ratios (0–0.2), hydrogen permeance decreased and the permeance ratio of H_2_/N_2_ increased with an increase in the Al/Si ratio, which confirmed that Al doping induces a densification of the pore structure. This is consistent with the results of N_2_ adsorption experimentation using BTESE- and Al-BTESE-derived powders. The second effect was an improvement in hydrophilicity. Water adsorption isotherms at room temperature showed a larger volume of adsorption for Al-BTESE-derived powder compared with that of the BTESE-derived version, even though N_2_ adsorption at −196 °C was greater for BTESE-derived powder than for the Al-BTESE-derived version, which confirmed the hydrophilic structure of the Al-BTESE-derived gel. In addition, H_2_O-TPD experimentation showed a significant level of water desorption from Al-BTESE-derived powder compared with that of the BTESE-derived version, which again confirmed improvement in the hydrophilicity *via* Al doping.

As for humid-gas separation (H_2_O/H_2_, H_2_O/N_2_), both BTESE- and Al-BTESE-derived membranes showed stable performance even under water vapor pressure as high as 360 kPa (abs) at 150 °C, which confirmed the hydrothermal stability. *Via* Al doping, the permeance ratios of H_2_O/H_2_ and H_2_O/N_2_ improved and reached several-tens and several-hundreds, respectively, while the high levels of water permeance were retained above 10^−6^ mol (m^−2^ s^−1^ Pa^−1^). This improved performance was concluded to be an outcome of increased molecular sieving and a blocking effect to prevent the permeation of non-condensable gases, which were results of a densified pore structure and enhanced hydrophilicity, respectively.

## Conflicts of interest

The authors declare that they have no competing interests.

## Supplementary Material

RA-012-D1RA07866F-s001
