# Supplementary material for: Network tailoring of organosilica membranes via aluminum doping to improve the humid-gas separation performance
Source: RSC Adv. 2022 Feb 16;12(10):5834–46. doi: 10.1039/d1ra07866f (PMC8981573; doi:10.1039/d1ra07866f)
Supplement: RA-012-D1RA07866F-s001 [file RA-012-D1RA07866F-s001.pdf]

Electronic Supplementary Material (ESI) for RSC Advances.
This journal is © The Royal Society of Chemistry 2022

Supporting Information (ESI†)

Network tailoring of organosilica membranes via aluminum doping to improve the humid-gas separation performance

Norihiro Moriyama, Misato Ike, Hiroki Nagasawa, Masakoto Kanezashi, and Toshinori Tsuru

Department of Chemical Engineering, Hiroshima University, 1-4-1 Kagami-yama, Higashi-Hiroshima 739-8527, Japan

---

Corresponding author: Toshinori Tsuru (tsuru@hiroshima-u.ac.jp)

Keywords: Steam; Humid gas; Organosilica membrane; Vapor permeation; Metal doping

---

Table of contents

ESI-1 Cross-sectional SEM images of membranes

ESI-2 Estimation of activation energy for permeation

ESI-3 XRD patterns

ESI-4 Nitrogen and water adsorption properties

ESI-5 Estimation of pore size via the normalized Knudsen-based (NKP) method

ESI-6 A schematic of permeation through pores consisting of amorphous networks and pinholes

ESI-7 Single-gas permeation properties

ESI-8 The effect of Al doping to improve the blocking effect

ESI-9 Water-contact angle

ESI-10 Time course of mass intensity and temperature during TPD measurements

ESI-11 Characterization of Al doping via NMR

ESI-12 Comparison of the humid-gas separation performances of membranes with various materials

ESI-1 Cross-sectional SEM images of membranes

Fig. S-1 shows cross-sectional SEM images of the BTESE- and Al-BTESE (Al/Si=0.1)-derived membranes. For both membranes, dense separation and intermediate layers were continuously formed on the supports with thicknesses of several hundred nm.

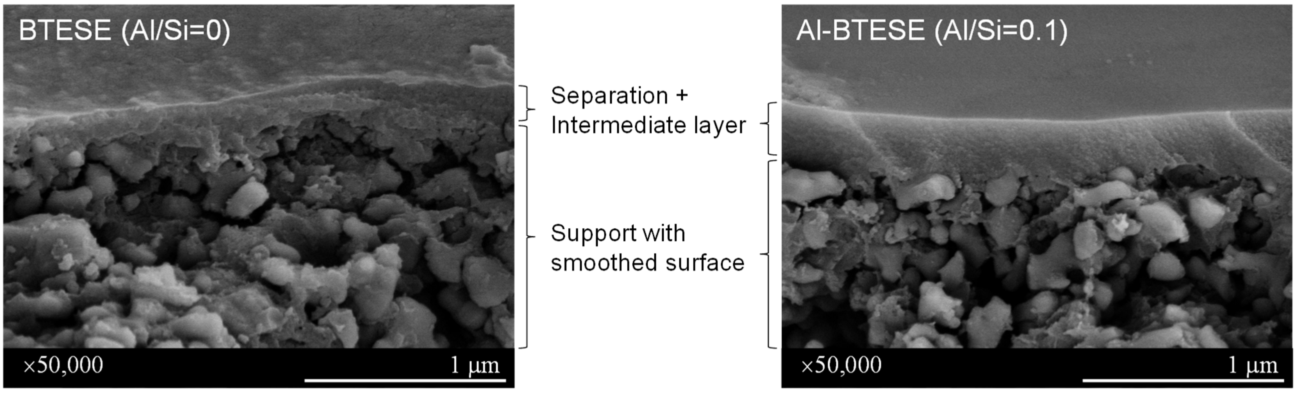

Fig. S-1 Cross-sectional SEM images of BTESE- and Al-BTESE (Al/Si=0.1)-derived membranes.

ESI-2 Estimation of activation energy for permeation

We proposed a modified gas translation (GT) model[1] expressed as Eq. (S-1), that was derived by modifying the original GT model proposed by Xiao and Wei [2] and by Shelekhin *et al.*[3] for membranes with pore sizes of less than 1 nm.

$$P_i = \frac{a}{\sqrt{M_i RT}}(d_0 - d_i)^3 \exp\left(-\frac{E_{P,i}}{RT}\right) \qquad \text{Eq. (S-1)}$$

In Eq. (S-1), P_i [mol/(m^2 s Pa)], M_i [g/mol], R [J/(mol K)], T [K], d_0 [nm], d_i [nm], and $E_{P,i}$ [J/mol] are the permeance, molecular weight, gas constant, temperature, pore size, molecular size, and activation energy for permeation, respectively.

Below, a [nm^{-3}] is a constant that depends only on membrane structures and is independent of the permeating gas species, as expressed in Eq. (S-2).

$$a = \frac{\varepsilon}{3\tau L d_o^2}\sqrt{\frac{8}{\pi}} \qquad \text{Eq. (S-2)}$$

In Eq. (S-2), ε [-], τ [-], L [nm], and d_0 [nm] are porosity, tortuosity, thickness, and pore size of the membrane.

By regressing each P_i to Eq. (S-1), $E_{P,i}$ can be obtained together with the value of $a\,(d_0 - d_i)^3$.

Fig. S-2 shows the temperature dependency of hydogen (a) and nitrogen (b) permeance through M-0, M-0.05, M-0.1 and M-0.2. All membranes showed a larger permeance of hydrogen and nitrogen at higher temperature, which indicates activation diffusion. The values for estimated activation energy are summarized in Table S-1. As the Al/Si molar ratio increased from 0 to 0.2, E_{P,H_2} increased from

2.2 to 8.9 kJ/mol. This means that a membrane with higher Al/Si has a larger energy requirement to overcome the barrier of hydrogen permeation, which indicates the densification of the membrane structure via Al-doping. In addition, as a general trend, E_{P,N_2} also increased as Al/Si increased, again confirming the structural densification. The slightly smaller E_{P,N_2} of M-0.2 than that of M-0.1 can be resonably explained by pinholes in M-0.2 where activation energy for permeation through pinholes is almost zero.

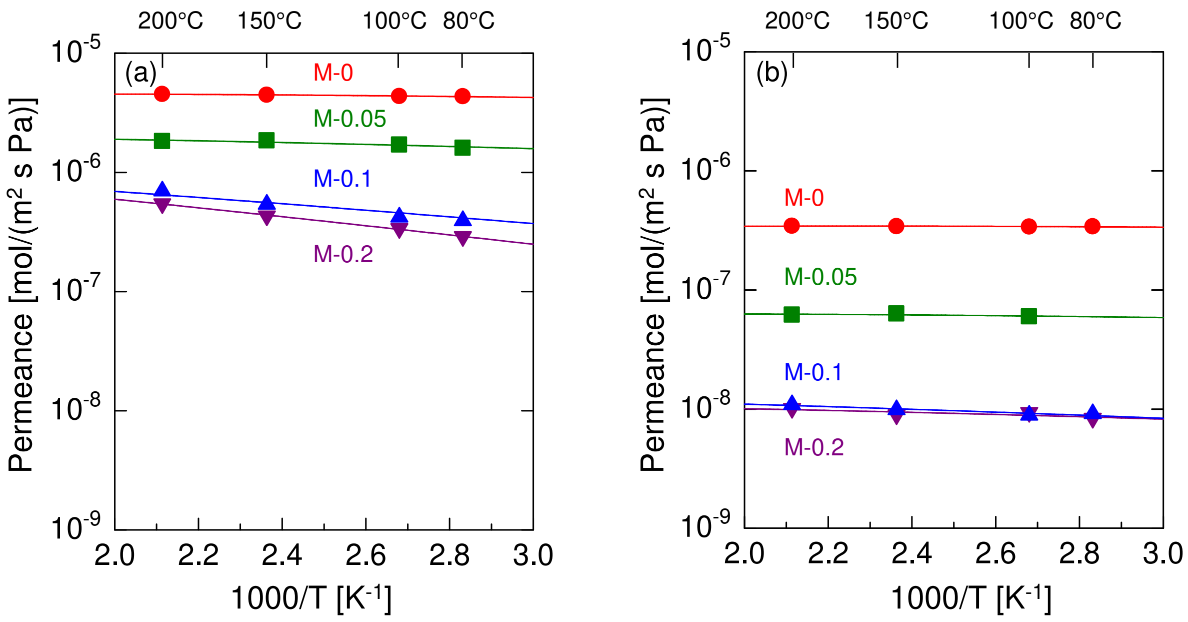

Fig. S-2 Temperature dependency of hydrogen (a) and nitrogen (b) permeance through M-0, M-0.05, M-0.1 and M-0.2. Symbols and lines indicate experimental results and fitted curves using Eq. (S-1), respectively. The estimated values of $E_{P,i}$ and α are summarized in Table S-1.

Table S-1 Values of $E_{P,i}$ and a for M-0, M-0.05, M-0.1, and M-0.2.

Membrane	Al/Si [-]	$a_{H_2}\left(d_0 - d_{H_2}\right)^3$ [-]	E_{P,H_2} [kJ/mol]	$a_{N_2}\left(d_0 - d_{N_2}\right)^3$ [-]	E_{P,N_2} [kJ/mol]
M-0	0	2.2×10^{-5}	2.2	5.7×10^{-6}	1.8
M-0.05	0.05	1.2×10^{-5}	3.2	1.2×10^{-6}	2.3
M-0.1	0.1	1.0×10^{-5}	6.9	3.1×10^{-7}	4.0
M-0.2	0.2	1.5×10^{-5}	8.9	2.5×10^{-6}	3.4

ESI-3 XRD patterns

Fig. S-3 shows the XRD patterns for BTESE- (Al/Si=0) and Al-BTESE-derived (Al/Si=0.05-0.2) powders. All types of powders showed broad peaks centered around 12 and 20-22° without any sharp peaks, confirming that no crystallization of Al occurred in the organosilica networks. The broad peaks can be assigned to the amorphous structure that typically originates from the Si-Si distance.[4] The peaks slightly shifted to high 2θ following an increase in the Al/Si ratio, which is due to the densification of amorphous pore structures via Al doping as confirmed by nitrogen adsorption (Fig. S-4) and single-gas permeation measurements (Fig. 5, Main Manuscript).

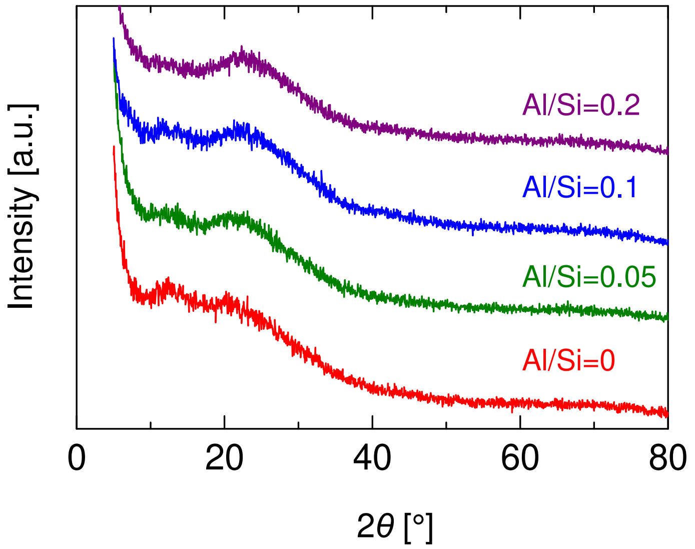

Fig. S-3 XRD patterns for BTESE- (Al/Si=0) and Al-BTESE-derived (Al/Si=0.05-0.2) powders.

ESI-4 Nitrogen and water adsorption properties

Fig. S-4 shows nitrogen adsorption and desorption isotherms for BTESE- (Al/Si=0) and Al-BTESE-derived (Al/Si=0.05-0.2) powders at -196°C. For all powders, the adsorption amount dramatically increased with a low p/p_s range and achieved values larger than 100 cm^3(STP)/g at p/p_s of approximately 1, confirming microporous structures. The adsorbed amount tended to decrease as Al/Si ratio decreased, which suggested a densification of the pore structure via Al doping.

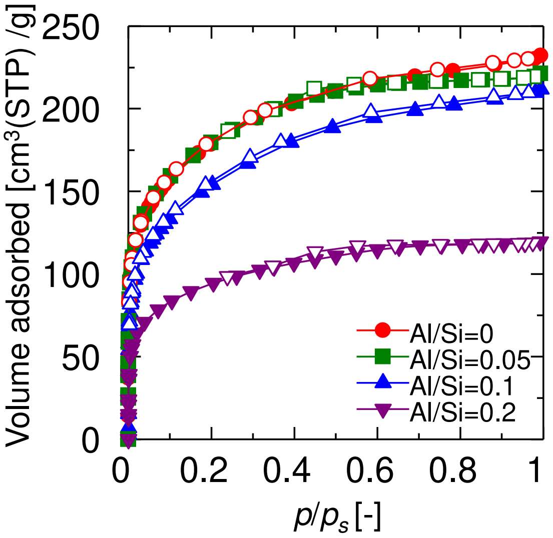

Fig. S-4 Nitrogen adsorption and desorption isotherms for BTESE- (Al/Si=0) and Al-BTESE-derived (Al/Si=0.05-0.2) powders at -196°C. The closed and open symbols indicate the levels of adsorption and desorption, respectively.

Fig. S-5 shows water adsorption and desorption isotherms for BTESE- (Al/Si=0) and Al-BTESE-derived (Al/Si=0.05-0.2) powders at 25°C (Al/Si=0, 0.05 (a), Al/Si =0.1, 0.2 (b)). The maximum adsorbed amounts (at p/p_s of approximately 1) were 350-400 cm^3(STP)/g for all powders and they tended to decrease following an increase in the Al/Si ratio. This trend is similar to the result of nitrogen adsorption and would be due to the more densified pore structure with higher Al content. On the other hand, based on the adsorbed amount within a low range of p/p_s (< 0.2), the adsorbed amount tended to increase together with the Al/Si ratio, suggesting improved hydrophilicity via Al doping. In addition, the hysteresis in this p/p_s range was more significant for a high Al content of powders (Fig. S-5 (b)) than for low content (Fig. S-5 (a)), which also indicates an improvement in hydrophilicity via Al doping.

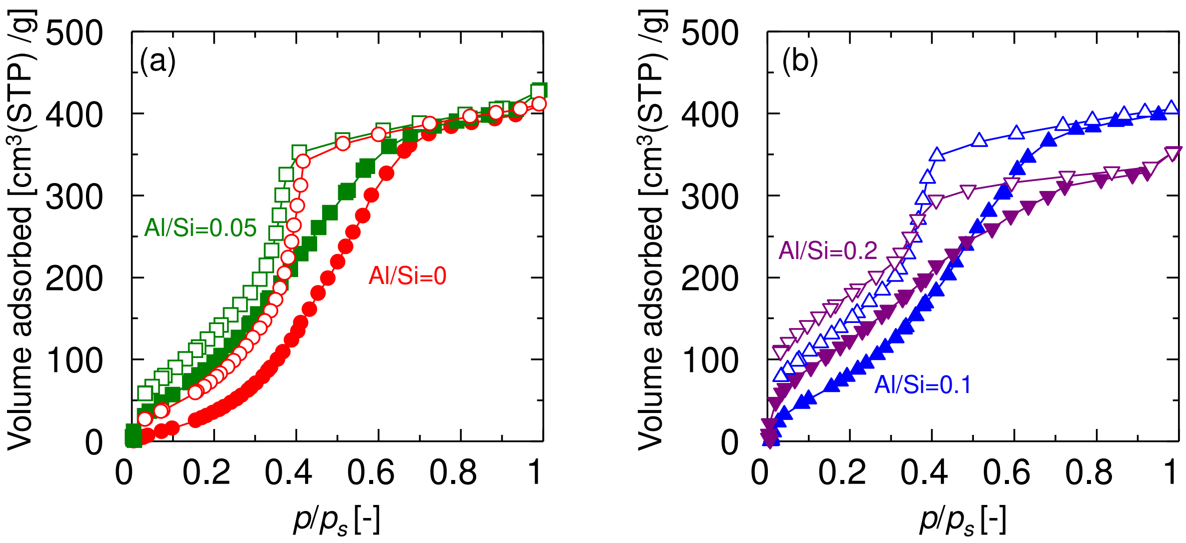

Fig. S-5 Water adsorption and desorption isotherms for BTESE- (Al/Si=0) and Al-BTESE-derived (Al/Si=0.05-0.2) powders at 25°C (Al/Si=0, 0.05 (a), Al/Si =0.1, 0.2 (b)). The closed and open symbols indicate the levels of adsorption and desorption, respectively.

ESI-5 Estimation of pore size via the normalized Knudsen-based (NKP) method

Eq. (S-1) can be converted as Eq. (S-3).

$$\left(\sqrt{M_i}P_i\right)^{\frac{1}{3}} = \left(\frac{a}{\sqrt{RT}}\exp\left(-\frac{E_{P,i}}{RT}\right)\right)^{\frac{1}{3}}(d_0 - d_i) \qquad \text{Eq.}\,(S-3)$$

When activation energies for each molecule can be assumed as a constant for simplicity, pore size, d_0, can be estimated by regressing each $\left(\sqrt{M_i}P_i\right)^{\frac{1}{3}}$ to Eq. (S-3).

Fig. S-6 shows the relationship between $\left(\sqrt{M_i}P_i\right)^{\frac{1}{3}}$ and the molecular size of permeating molecules at 200°C. The results of small gases such as He, H_2, N_2, and CH_4 were plotted on the same line, which indicates that they mainly permeated through the same pores. According to Eq. (S-3), the intercept of molecular size at $\left(\sqrt{M_i}P_i\right)^{\frac{1}{3}} = 0$ correponds to the pore size. This analysis confirmed that the pore size decreased from 0.56 to 0.42 nm as the Al/Si ratio was increased from 0 to 0.2. Of note, large gases such as CF_4 and SF_6 deviated from the regressed lines, suggesting that they mainly permeated limited numbers of large pores such as pinholes.

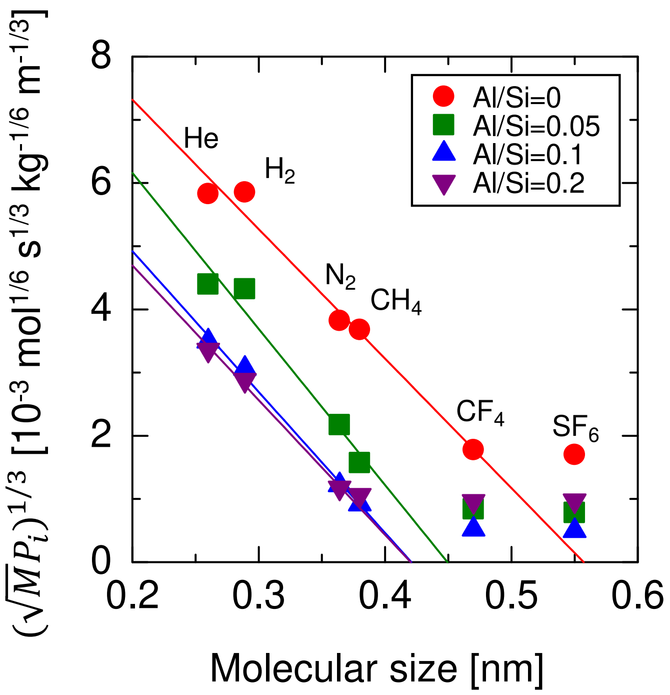

Fig. S-6 Relationship between $\left(\sqrt{M_i}P_i\right)^{\frac{1}{3}}$ and molecular size of permeating molecules at 200°C. Symbols and lines indicate experimental results and fitted lines, respectively.

ESI-6 A schematic of permeation through pores consisting of amorphous networks and pinholes

Fig. S-7 illustrates gas permeation through an organosilica membrane with a limited number of pinholes. Pores formed by the organosilica network allow permeation of small gas but prevent that of large gas via molecular sieving. On the other hand, pinholes allow permeation of all gases via Knudsen diffusion. The permeance of small gases such as He and H_2 are not affected by a limited number of pinholes because they mainly permeate through network pores. However, even if the number of pinholes is limited, they dominate, and increase, the permeance of large gases such as CF_4 and SF_6 because these gases cannot permeate the network pores.

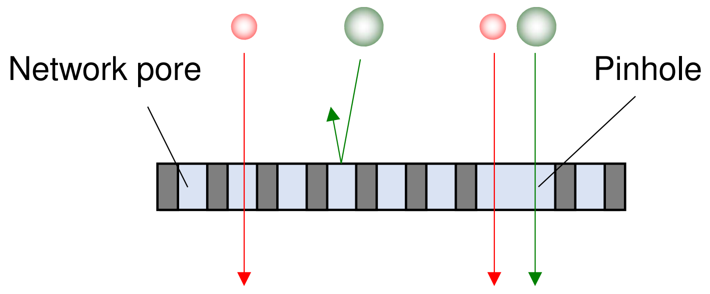

Fig. S-7 Schematic of gas permeation through an organosilica membrane with a limited number of pinholes.

ESI-7 Single-gas permeation properties

Fig. S-8 shows single-gas permeances and permeance ratios as a function of the Al/Si ratio. As Al/Si increased the permeance of hydrogen and nitrogen decreased and H_2/N_2 increased, which confirmed densification of the organosilica networks via Al-doping. The permeance of SF_6 also decreased when Al/Si increased from 0 to 0.1 but increased at Al/Si of 0.2. This is probably due to the generation of non-negligible numbers of large pores such as pinholes when Al/Si=0.2, as schematically shown in ESI-6, which were probably caused by the micrometer-ordered particles formed in the Al/Si=0.2 sol due to the saturation of Al into the BTESE-derived structure. As a result, the value of H_2/SF_6 decreased with an Al/Si of 0.2.

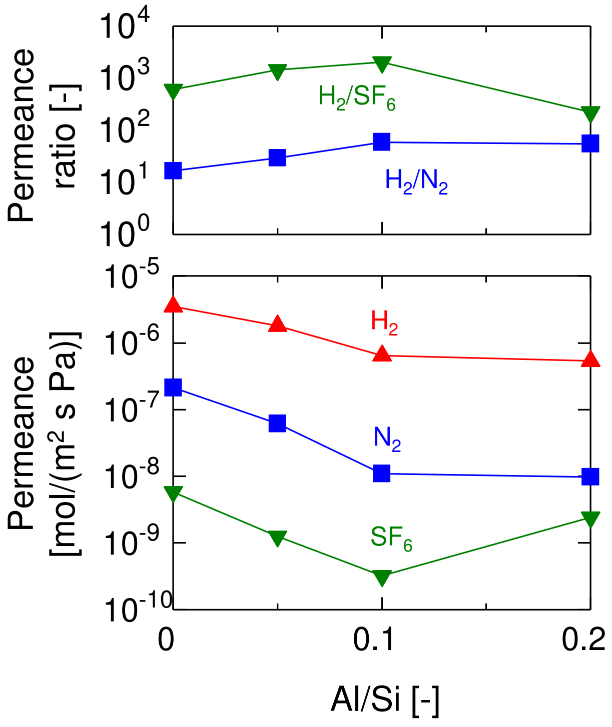

Fig. S-8 Single-gas permeances of hydrogen, nitrogen and sulfur hexafluoride at 200°C and the permeance ratios as a function of the Al/Si ratio (M-0, M-0.05, M-0.1 and M-0.2).

ESI-8 The effect of Al-doping to improve the blocking effect

Fig. S-9 shows the molecular size dependency of single-gas permeance through BTESE- (M-0 and M-0*) and Al-BTESE-derived membranes (M-0.05, M-0.1 and M-0.2) at 200°C. The details of the preparation conditions for each membrane can be found in Table 1 (Main Manuscript). The H_2/N_2 permeance ratio of M-0* (BTESE-derived) was 42, which was comparable to those of Al-BTESE-derived membranes at a range of from 29 to 59. This indicates that the pore size of M-0* was comparable to that of Al-derived membranes in spite of the Al/Si ratio of zero.

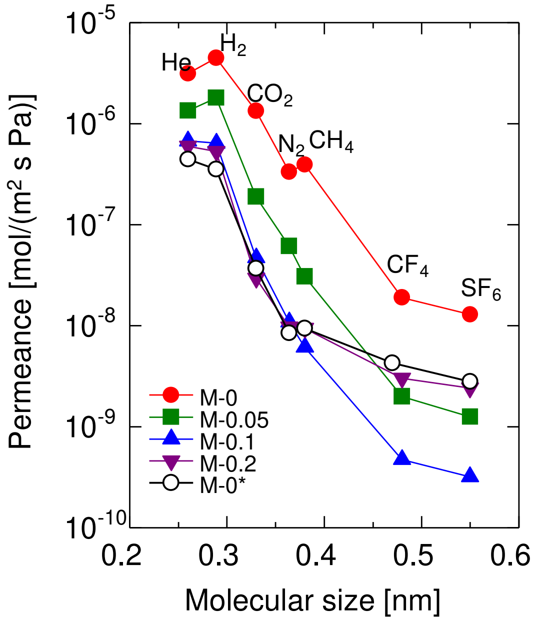

Fig. S-9 Molecular size dependency of single-gas permeance at 200°C for M-0, M-0.05, M-0.1, M-0.2 and M-0*. The details of the preparation conditions for each membrane can be found in Table 1 (Main Manuscript).

Fig. S-10 shows the relationship between $P_{H_2,wet}/P_{H_2,dry}$ (150°C, x_w=0.5, feed: 400 kPa-a; permeate: atmospheric pressure) and the single-gas permeance ratio of H_2/N_2 where H_2/N_2 is an index of the pore size. Obviously, the $P_{H_2,wet}/P_{H_2,dry}$ of Al-BTESE-derived membranes were smaller than that of BTESE-derived membranes regardless of the value of H_2/N_2. This clarified that Al-doping was effective to improve the blocking effect.

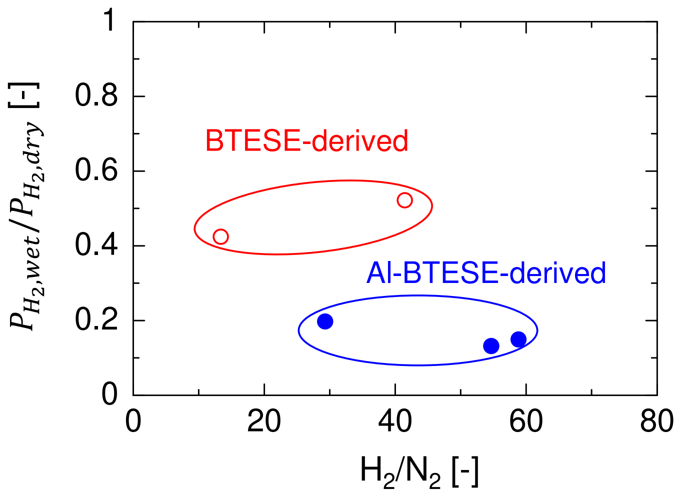

Fig. S-10 The relationship between $P_{H_2,wet}/P_{H_2,dry}$ (150°C, x_w=0.5, feed: 400 kPa-a; permeate: atmospheric pressure) and the single-gas permeance ratio of H_2/N_2. The open and closed symbols indicate BTESE- and Al-BTESE-derived membranes, respectively.

ESI-9 Water contact angle

Measurement of the static water-contact angle was performed on BTESE- and Al-BTESE-derived films prepared by coating sols onto Si wafers and fired at 300°C under a nitrogen flow. A Dropmaster (DM-300, Kyowa Co., Japan) was used for this measurement. Fig. S-11 shows the static water-contact angle against the Al/Si ratio. As the Al/Si ratio was increased from 0 to 0.2, the water-contact angle decreased from 74 to 52°, which indicates Al doping improved the hydrophilicity of the BTESE-derived gels.

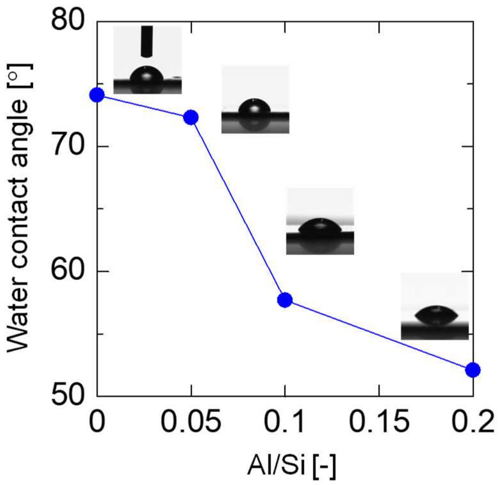

Fig. S-11 Static water-contact angle on BTESE- (Al/Si=0) and Al-BTESE-derived (Al/Si=0.05-0.2) films against the Al/Si ratio.

ESI-10 Time course of mass intensity and temperature during TPD measurements

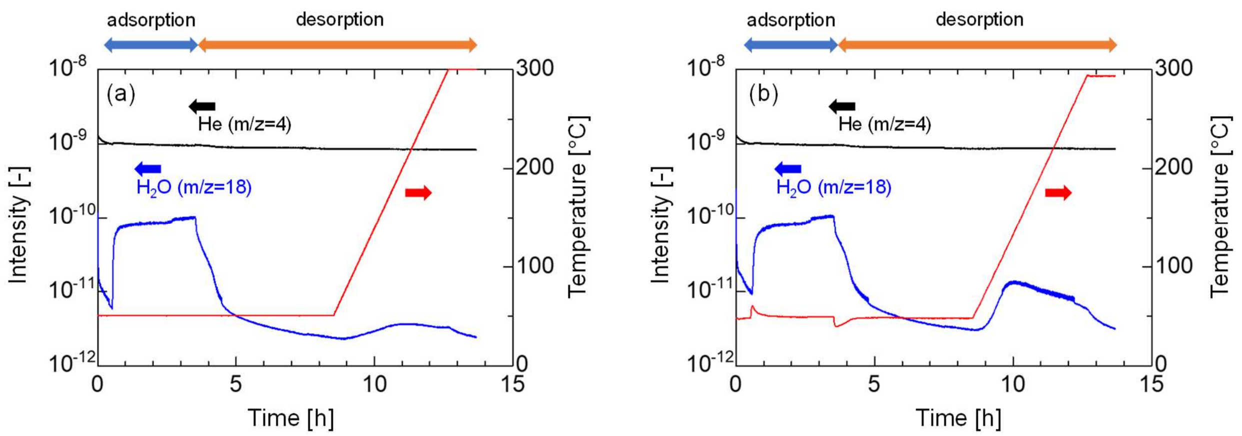

Fig. S-12 Time course of mass intensity and temperature during H_2O-TPD measurements of BTESE-(Al/Si=0) (a) and Al-BTESE-derived (Al/Si=0.1) powders (b).

ESI-11 Characterization of Al doping via NMR

BTESE- and Al-BTESE-derived powders were analyzed via nuclear magnetic resonance (NMR, Varian 600PS solid NMR spectrometer). Fig. S-13 shows the ^{27}Al MAS NMR spectra of BTESE- and Al-BTESE-derived (Al/Si=0.1) powders. The Al-BTESE-derived powder showed two peaks, which confirmed the incorporation of aluminum into organosilica networks, while no peaks were observed for the BTESE-derived versions. Moreover, the two peaks indicated that aluminum was incorporated in two different states. The first peak centered around 60ppm was assigned to tetrahedral Al, which indicated the $Al(OSi)_4$ framework, while the second peak centered around 0ppm was due to octahedral Al, which could be Al^{3+} ions coordinated with silanol groups and water molecules.[5, 6] This result is similar to our previous report that evaluated Al doping into bis(triethoxysilyl)methan (BTESM)-derived organosilica networks.[7]

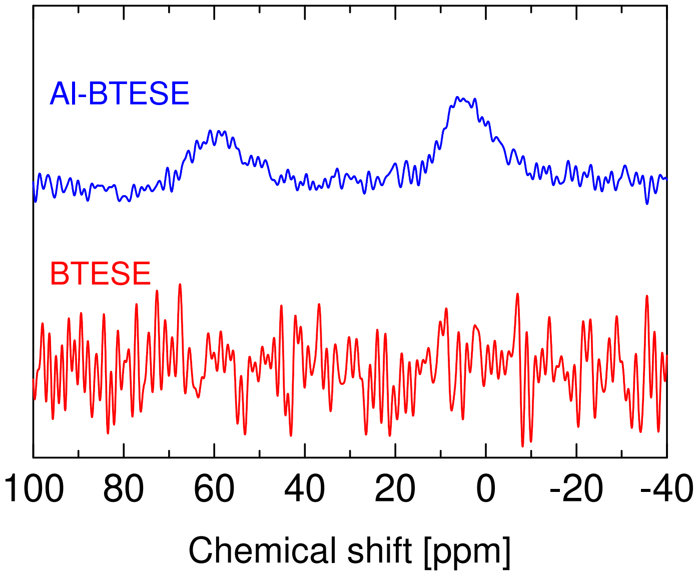

Fig. S-13 ^{27}Al MAS NMR spectra of BTESE- and Al-BTESE-derived (Al/Si=0.1) powders.

ESI-12 Comparison of the humid-gas separation performances of membranes with various materials

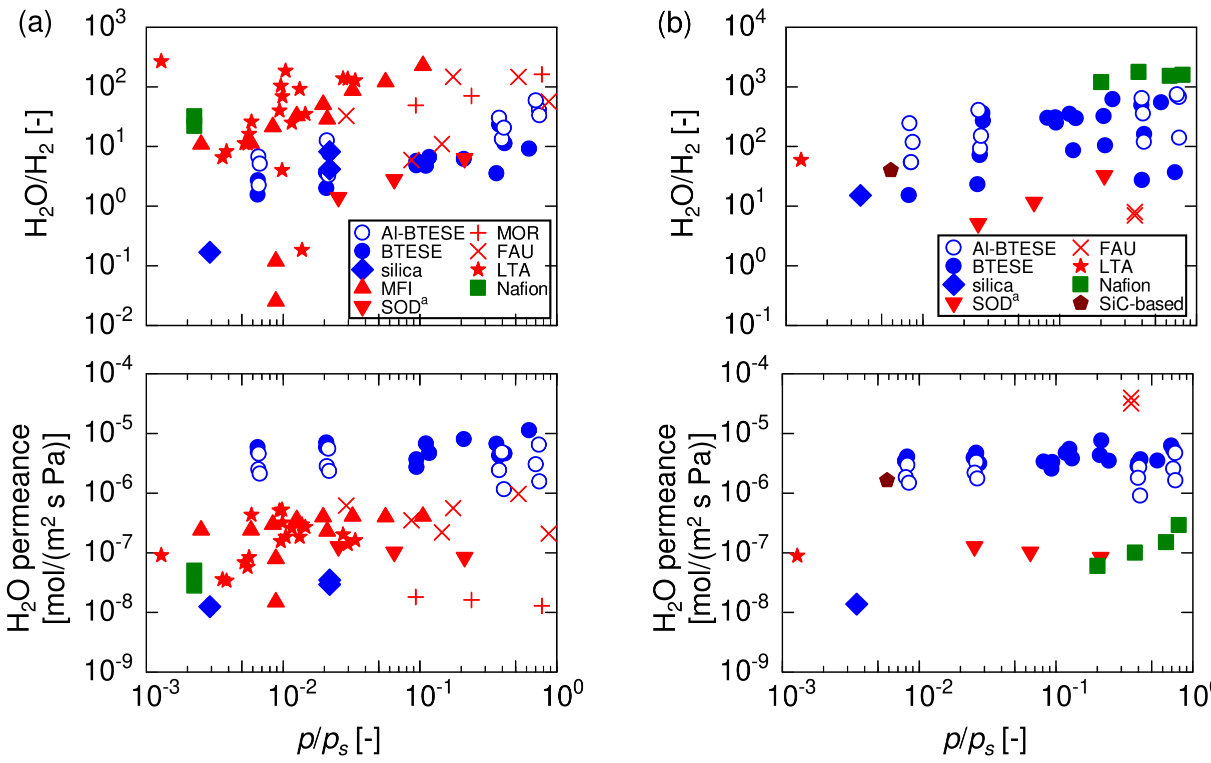

Fig. S-14 Permeances and permeance ratios of H_2O/H_2 (a) and H_2O/N_2 (b) as a function of relative pressure p/p_s at 150-300°C, for references, please see Table S-2. [a]Performance in single-gas permeation.

Table S-2 Humid gas separation performance of membranes at 150-300°C; symbols refer to Figs. 15 (Main Manuscript) and S-6.

Material	System	Temperature [°C]	Water vapor pressure in feed [kPa-a]	p/p_s×10^2 [-]	Water permeance ×10^8 [mol/(m^2 s Pa)]	Permeance ratio [-] H_2O/H_2	Permeance ratio [-] H_2O/N_2	Reference
Al-BTESE	H_2O/H_2	200	10	0.67	460	2.2	-	This work
Al-BTESE	H_2O/H_2	150	10	2.1	550	3.3	-	This work
Al-BTESE	H_2O/H_2	150	190	40	480	13	-	This work
Al-BTESE	H_2O/H_2	150	353	74	650	42	-	This work
Al-BTESE	H_2O/H_2	200	10	0.66	250	6.7	-	This work
Al-BTESE	H_2O/H_2	150	10	2.1	280	13	-	This work

Al-BTESE	H_2O/H_2	150	181	38	240	30	-	This work
Al-BTESE	H_2O/H_2	150	335	70	300	59	-	This work
Al-BTESE	H_2O/H_2	200	11	0.68	310	5.1	-	This work
Al-BTESE	H_2O/H_2	150	10	2.2	230	7.7	-	This work
Al-BTESE	H_2O/H_2	150	196	41	120	20	-	This work
Al-BTESE	H_2O/H_2	150	356	75	160	33	-	This work
Al-BTESE	H_2O/N_2	200	13	0.82	300	-	54	This work
Al-BTESE	H_2O/N_2	150	12	2.6	330	-	92	This work
Al-BTESE	H_2O/N_2	150	193	41	270	-	360	This work
Al-BTESE	H_2O/N_2	150	354	74	470	-	670	This work
Al-BTESE	H_2O/N_2	200	12	0.80	180	-	240	This work
Al-BTESE	H_2O/N_2	150	12	2.6	220	-	410	This work
Al-BTESE	H_2O/N_2	150	190	40	180	-	640	This work
Al-BTESE	H_2O/N_2	150	343	72	260	-	740	This work
Al-BTESE	H_2O/N_2	200	13	0.85	150	-	120	This work
Al-BTESE	H_2O/N_2	150	13	2.7	180	-	150	This work
Al-BTESE	H_2O/N_2	150	197	41	90	-	120	This work
Al-BTESE	H_2O/N_2	150	356	75	160	-	140	This work
BTESE	H_2O/H_2	200	10	0.66	490	2.7	-	[8]
BTESE	H_2O/H_2	150	10	2.1	580	3.6	-	[8]
BTESE	H_2O/H_2	150	10	2.1	590	3.6	-	[8]
BTESE	H_2O/H_2	150	53	11	680	4.7	-	[8]
BTESE	H_2O/H_2	150	100	21	800	6.2	-	[8]
BTESE	H_2O/H_2	200	182	12	470	6.6	-	[8]
BTESE	H_2O/H_2	200	147	9.4	370	4.8	-	[8]
BTESE	H_2O/H_2	150	198	42	460	11	-	[8]
BTESE	H_2O/H_2	200	147	9.5	270	5.7	-	[8]
BTESE	H_2O/H_2	200	10	0.65	590	1.6	-	This work
BTESE	H_2O/H_2	150	10	2.1	700	2.0	-	This work
BTESE	H_2O/H_2	150	173	36	670	3.5	-	This work
BTESE	H_2O/H_2	150	299	63	1100	9.1	-	This work
BTESE	H_2O/H_2	150	181	38	430	23	-	This work
BTESE	H_2O/N_2	200	13	0.82	410	-	54	[8]
BTESE	H_2O/N_2	150	12	2.6	450	-	71	[8]
BTESE	H_2O/N_2	150	12	2.6	470	-	73	[8]
BTESE	H_2O/N_2	150	60	13	550	-	86	[8]
BTESE	H_2O/N_2	150	102	22	760	-	100	[8]

BTESE	H_2O/N_2	200	184	12	470	-	350	[8]
BTESE	H_2O/N_2	200	146	9.4	330	-	250	[8]
BTESE	H_2O/N_2	150	197	42	370	-	160	[8]
BTESE	H_2O/N_2	200	145	9.3	260	-	310	[8]
BTESE	H_2O/N_2	150	13	2.8	310	-	270	[9]
BTESE	H_2O/N_2	150	39	8.1	340	-	300	[9]
BTESE	H_2O/N_2	150	62	13	380	-	300	[9]
BTESE	H_2O/N_2	150	100	21	430	-	320	[9]
BTESE	H_2O/N_2	150	13	2.7	320	-	360	[9]
BTESE	H_2O/N_2	150	116	24	350	-	620	[9]
BTESE	H_2O/N_2	150	189	40	280	-	490	[9]
BTESE	H_2O/N_2	150	263	55	350	-	550.0	[9]
BTESE	H_2O/N_2	150	343	72	530	-	710	[9]
BTESE	H_2O/N_2	200	12	0.79	340	-	15	This work
BTESE	H_2O/N_2	150	12	2.5	390	-	23	This work
BTESE	H_2O/N_2	150	190	40	320	-	27	This work
BTESE	H_2O/N_2	150	332	70	620	-	37	This work
Slica	$H_2O/H_2/N_2/CO_2/CO$	200	34	2.2	3.5	8.2	-	[10]
Slica	$H_2O/H_2/N_2/CO_2/CO$	200	34	2.2	3.0	4.2	-	[10]
Slica	H_2O/H_2	300	25	0.29	1.3	0.17	-	[11]
Slica	H_2O/N_2	300	30	0.35	1.4	-	15	[11]
MFI	H_2O/H_2	150	50	11	41	230	-	[12]
MFI	H_2O/H_2	175	50	5.6	40	120	-	[12]
MFI	H_2O/H_2	200	50	3.2	41	87	-	[12]
MFI	H_2O/H_2	225	50	2.0	40	51	-	[12]
MFI	H_2O/H_2	250	50	1.3	36	33	-	[12]
MFI	H_2O/H_2	275	50	0.84	30	21	-	[12]
MFI	H_2O/H_2	300	50	0.58	24	11	-	[12]
MFI	H_2O/H_2	150	10	2.1	23	29	-	[12]
MFI	H_2O/H_2	250	10	0.25	24	11	-	[12]
MFI	$H_2O/H_2/CO_2$	300	76	0.89	1.5	0.025	-	[13]
MFI	$H_2O/H_2/CO_2$	300	76	0.89	8.0	0.12	-	[13]
SOD	Single-gas	150	101	21	8.4	6.3	33	[14]
SOD	Single-gas	200	101	6.5	10	2.8	12	[14]
SOD	Single-gas	250	101	2.5	13	1.4	5.1	[14]

MOR	H_2O/H_2/MeOH	150	370	78	1.3	160	-	[15]
MOR	H_2O/H_2/MeOH	200	370	24	1.6	71	-	[15]
MOR	H_2O/H_2/MeOH	250	370	9.3	1.8	49	-	[15]
FAU	H_2O/H_2/MeOH	180	175	17	56	150	-	[16]
FAU	H_2O/H_2/MeOH	180	525	52	97	150	-	[16]
FAU	H_2O/H_2/MeOH	180	875	87	21	57	-	[16]
FAU	H_2O/H_2/MeOH	180	29	2.9	62	33	-	[16]
FAU	H_2O/H_2/MeOH	180	87	8.7	35	5.9	-	[16]
FAU	H_2O/H_2/MeOH	180	145	14	22	11	-	[16]
FAU	H_2O/N_2	200	550	35	3200	-	8.0	[17]
FAU	H_2O/N_2	200	550	35	4000	-	7.0	[17]
LTA	H_2O/H_2	240	46	1.4	30	0.18	-	[18]
LTA	H_2O/H_2	200	2.0	0.13	9.1	270	-	[19]
LTA	$H_2O/H_2/CO/CO_2$/ MeOH	200	6.0	0.39	3.4	8.3	-	[20]
LTA	$H_2O/H_2/CO/CO_2$/ MeOH	210	6.9	0.36	3.6	6.5	-	[20]
LTA	$H_2O/H_2/CO/CO_2$/ MeOH	220	13	0.55	5.8	11	-	[20]
LTA	$H_2O/H_2/CO/CO_2$/ MeOH	230	15	0.52	6.9	11	-	[20]
LTA	$H_2O/H_2/CO/CO_2$/ MeOH	240	19	0.56	8.4	16	-	[20]
LTA	$H_2O/H_2/CO/CO_2$/ MeOH	200	43	2.7	20	140	-	[21]
LTA	$H_2O/H_2/CO/CO_2$/ MeOH	200	52	3.4	16	130	-	[21]
LTA	$H_2O/H_2/CO/CO_2$/ MeOH	250	38	0.96	16	100	-	[21]
LTA	$H_2O/H_2/CO/CO_2$/ MeOH	250	53	1.3	19	92	-	[21]
LTA	$H_2O/H_2/CO/CO_2$/ MeOH	250	41	1.0	19	190	-	[21]
LTA	$H_2O/H_2/CO/CO_2$/ MeOH	250	118	3.0	14	140	-	[21]
LTA	$H_2O/H_2/CO/CO_2$/ MeOH	250	46	1.2	26	25	-	[21]

LTA	$H_2O/H_2/CO/CO_2/$ MeOH	250	58	1.5	27	35	-	[21]
LTA	$H_2O/H_2/CO/CO_2/$ MeOH	250	39	0.98	53	4.0	-	[21]
LTA	$H_2O/H_2/CO/CO_2/$ MeOH	250	23	0.59	43	26	-	[21]
LTA	$H_2O/H_2/CO/CO_2/$ MeOH	250	39	0.99	32	69	-	[21]
LTA	$H_2O/H_2/CO/CO_2/$ MeOH	250	37	0.94	51	40	-	[21]
LTA	H_2O/N_2	200	2.0	0.13	8.9	-	59	[19]
Nafion	$H_2O/H_2/CO_2$	200	3.5	0.23	2.8	22	-	[22]
Nafion	$H_2O/H_2/CO_2$	200	3.5	0.23	5.0	32	-	[22]
Nafion	H_2O/N_2	150	96	20	6.0	-	1200	[23]
Nafion	H_2O/N_2	150	180	38	10	-	1800	[23]
Nafion	H_2O/N_2	150	304	64	15	-	1500	[23]
Nafion	H_2O/N_2	150	377	79	29	-	1600	[23]
SiC-based	H_2O/N_2	300	50	0.58	160	-	40	[24]

1. H. R. Lee, M. Kanezashi, Y. Shimomura, T. Yoshioka and T. Tsuru, *AlChE J.*, 2011, **57**, 2755-2765.
2. J. Xiao and J. Wei, *Chem. Eng. Sci.*, 1992, **47**, 1123-1141.
3. A. B. Shelekhin, A. G. Dixon and Y. H. Ma, *AlChE J.*, 1995, **41**, 58-67.
4. U. Anggarini, L. Yu, H. Nagasawa, M. Kanezashi and T. Tsuru, *ACS Appl Mater Interfaces*, 2021, **13**, 23247-23259.
5. J. R. Houston, J. L. Herberg, R. S. Maxwell and S. A. Carroll, *Geochim. Cosmochim. Acta*, 2008, **72**, 3326-3337.
6. M. Müller, G. Harvey and R. Prins, *Microporous Mesoporous Mater.*, 2000, **34**, 281-290.
7. M. Kanezashi, S. Miyauchi, H. Nagasawa, T. Yoshioka and T. Tsuru, *RSC Adv.*, 2013, **3**, 12080-12083.
8. N. Moriyama, H. Nagasawa, M. Kanezashi and T. Tsuru, *J. Membr. Sci.*, 2021, **620**, 118895.
9. N. Moriyama, H. Nagasawa, M. Kanezashi and T. Tsuru, *Sep. Purif. Technol.*, 2021, **275**, 119191.
10. M. Duke, J. C. D. da Costa, G. Q. M. Lu, M. Petch and P. Gray, *J. Membr. Sci.*, 2004, **241**, 325-333.
11. T. Tsuru, R. Igi, M. Kanezashi, T. Yoshioka, S. Fujisaki and Y. Iwamoto, *AlChE J.*, 2011, **57**, 618-629.
12. K. Sawamura, T. Izumi, K. Kawasaki, S. Daikohara, T. Ohsuna, M. Takada, Y. Sekine, E. Kikuchi and M.

Matsukata, *Chem Asian J*, 2009, **4**, 1070-1077.

13. H. Wang and Y. S. Lin, *AlChE J.*, 2012, **58**, 153-162.
14. M. Lafleur, F. Bougie, N. Guilhaume, F. Larachi, P. Fongarland and M. C. Iliuta, *Microporous Mesoporous Mater.*, 2017, **237**, 49-59.
15. K.-I. Sawamura, T. Shirai, M. Takada, Y. Sekine, E. Kikuchi and M. Matsukata, *Catal. Today*, 2008, **132**, 182-187.
16. K. Sato, K. Sugimoto, Y. Sekine, M. Takada, M. Matsukata and T. Nakane, *Microporous Mesoporous Mater.*, 2007, **101**, 312-318.
17. T. F. Mastropietro, A. Brunetti, P. F. Zito, T. Poerio, H. Richter, M. Weyd, S. Wöhner, E. Drioli and G. Barbieri, *Sep. Purif. Technol.*, 2015, **156**, 321-327.
18. S. M. Lee, N. Xu, J. R. Grace, A. Li, C. J. Lim, S. S. Kim, F. Fotovat, A. Schaadt and R. J. White, *J. Eur. Ceram. Soc.*, 2018, **38**, 211-219.
19. K. Aoki, K. Kusakabe and S. Morooka, *Ind. Eng. Chem. Res.*, 2000, **39**, 2245-2251.
20. R. Raso, M. Tovar, J. Lasobras, J. Herguido, I. Kumakiri, S. Araki and M. Menéndez, *Catal. Today*, 2021, **364**, 270-275.
21. H. Li, C. Qiu, S. Ren, Q. Dong, S. Zhang, F. Zhou, X. Liang, J. Wang, S. Li and M. Yu, *Science*, 2020, **367**, 667-671.
22. R. P. W. J. Struis, S. Stucki and M. Wiedorn, *J. Membr. Sci.*, 1996, **113**, 93-100.
23. H. Azher, C. A. Scholes, G. W. Stevens and S. E. Kentish, *J. Membr. Sci.*, 2014, **459**, 104-113.
24. Q. Wang, L. Yu, H. Nagasawa, M. Kanezashi and T. Tsuru, *J. Am. Ceram. Soc.*, 2020, **103**, 4473-4488.
